# Modelling and simulation of the block pouring construction system considering spatial–temporal conflict of construction machinery in arch dams

**DOI:** 10.1038/s41598-025-09998-6

**Published:** 2025-08-02

**Authors:** Zhipeng Liang, Jiayao Peng, Chunju Zhao, Huawei Zhou, Dongfeng Li, Yihong Zhou, Quan Liu, Xiaodong Li, Cheng Zhang, Fang Wang

**Affiliations:** 1 Key Laboratory of Health Intelligent Perception and Ecological Restoration of River and Lake, Ministry of Education, Hubei University of Technology, Wuhan, China; 2 School of Civil Engineering, Architecture and Environment, Hubei University of Technology, Wuhan, China; 3 Sinohydro Bureau 3 Co, Ltd PowerChina, Xian, China; 4 School of Water Resources and Hydropower Engineering, Wuhan University, Wuhan, China

**Keywords:** Spatial–temporal conflict, Construction organization and management, Pouring construction simulation system considering spatial–temporal conflict, System simulation, Concrete arch dams, Civil engineering, Computational science

## Abstract

The spatial–temporal conflicts in the construction process may cause a series of construction quality, safety and schedule problems. The outbreak of mechanical spatial–temporal conflict in the construction process of the arch dam pouring block is random and uncertain. Scientific simulation and preview of the pouring construction process and analysis of the level, time, and influence degree of the outbreak of spatial–temporal conflict are significant means to optimize the construction organization and management. According to the degree of spatial–temporal conflict and its effect on security and efficiency, the subsidiary space scope of construction machinery is divided into three levels from inside to outside. The quantification algorithm of spatial–temporal conflict is proposed based on the three-layered space and time–space microelement model. The discrete system theory is employed to develop a simulation framework that systematically incorporates four core components: simulation objectives, construction machinery operational cycles, resource allocation mechanisms, and modeling assumptions. Combined with the typical pouring block in Baihetan arch dam, the construction process is simulated and the visualization system is developed, which achieves the information integration such as the quantification of the spatial–temporal conflict, the analysis of the influence effects, and the visualization of conflict information. The system simulation results show that the spatial–temporal conflict problem always exists in the pouring construction process, the problems of security risk and efficiency loss are inevitable. And the reasonable unloading point planning and mechanical trajectory setting can effectively reduce the risk of spatial–temporal conflict. Those studies provide a reference for the rational organization and scientific decision-making of pouring construction activities, and new ideas and methods for the safe and efficient construction, as well as the scientific and refined management, of arch dams.

## Introduction

### Research background

Concrete arch dams occupy an important position in the field of dam engineering around the world, of which pouring construction for the dam body is the core of ensuring quality and safety in arch dam construction and realizing continuous high-strength and rapid construction^1–4^. Concrete arch dams generally adopt a layered and block pouring method, and each pouring block forms a relatively independent unit. There are hundreds or thousands of pouring blocks in large-scale arch dam projects. From a systematic perspective, the construction process of arch dams is a discrete dynamic process formed by the pouring of each block in a specific order. The construction safety and efficiency of a dam are associated with the safety and efficiency of each pouring block^5–7^. The construction safety and efficiency are associated with the safety and efficiency of each pouring block for an arch dam. The safety and efficiency of construction activities are the core of construction organization and management, because they directly affect the quality and duration of dam construction^8–10^. Therefore, to ensure the quality and duration of concrete construction, the construction organization of a pouring block should be optimized to improve the construction efficienc^11–13^.

As the construction of water conservancy projects gradually moves in the direction of complexity, higher and more comprehensive requirements are being put forward for the scientific and refined management and control of the dam construction proces^14,15^. This has also prompted researchers to gradually divide the whole process of dam construction into deeper and more detailed construction procedures^16^. The dam construction process is a dynamic and continuous composite system. As an important basic unit of arch dam construction, block pouring construction is continuous and dynamic. The continuous, divided, and dynamically adjusted pouring block construction finally forms the overall structure of a dam. Moreover, the safety risks and efficiency losses arising from the single-block pouring construction process are introduced into the overall construction safety and construction efficiency of a dam. During the pouring construction process, the construction operations driven by the pouring construction process activities are carried out according to the specific setting process and their respective movement timelines. Mechanical equipment such as a concrete spreading machine (CSM), a concrete vibrating machine (CVM), and a cable machine are used for the main portion of pouring construction work. The volumes of these machines are relatively large and are limited by the relatively limited space in the pouring block. There are parallel construction activities during the construction process, and the same construction space may need to be occupied at the same time, which will inevitably lead to spatial–temporal conflict, which will in turn lead to physical collisions, safety risks, and efficiency losses.

The pouring construction system of the block is one of the smallest unit systems that constitute the overall construction of the arch dam, which has the characteristics of many construction disturbances, complex construction procedures, and narrow construction sites. Meanwhile, the structure of the pouring block is complex, including many corridors, holes, embedded parts, steel mesh et al. The unique construction characteristics and composition structure of the pouring construction system have formed a relatively mature construction process flow and a dynamic adjustment of the on-site construction organization mode. However, In the process of concrete pouring, factors such as complicated machinery and auxiliary equipment, as well as difficulties in the mechanical layout caused by space resource constraints, further aggravate the spatial–temporal conflict in the process of space resource allocation and utilization, resulting in safety risks or efficiency losses^17–19^. A spatial–temporal conflict is a situation in which labor crews assigned to two or more concurrent activities share a common workspace. When parallel construction activities occupy the same construction space at the same time, a spatial–temporal conflict occur^20^. The spatial–temporal conflicts significantly hinder the performance of overlapping activities and are a major source of construction labor productivity loss^21^.

However, the safety and efficiency analysis method based on experience guidance and index evaluation cannot meet the growing demand for refined and intelligent management. Especially for the complex and discrete construction system of the pouring blocks, the outbreak time of spatial–temporal conflict under the influence of multiple factors is random and accidental, which cannot be effectively obtained. Therefore, for the complex pouring construction system of the arch dams, it is of great significance to improve the scientific and refined management of pouring construction process by analyzing the time and influence degree of conflict outbreak, previewing and predicting space–time conflict and optimizing construction scheme through system simulation.

### Literature review

Due to the particularity of concrete arch dam construction and the many limitations, there are still few studies on the safety risks and efficiency losses caused by spatial–temporal conflicts in the construction process of a concrete arch dam. Hu et al.^22^ analyzed the generation mechanism of spatial–temporal conflicts according to the transition process of construction entities and studied the classification and influence effect of conflict. Omid et al.^23^ proposed an evaluation method based on Monte Carlo simulation and risk management indices, taking concrete dam projects as an example. Hidayah et al.^24^ developed a risk-based work breakdown structure (WBS) standard for safety planning in dam construction as an effort to prevent, reduce, or eliminate accidents in dam construction work. Wu et al.^25^ established a safety monitoring platform based on GNSS/GIS integration technology that achieved the analysis of safety conditions in cable crane hoisting construction. Hwang^26^ established a real-time monitoring method of the running status and collision risk for a cable crane utilizing ultra-wideband technology. Zhang et al.^27^ proposed a collision detection and real-time trajectory adjustment optimization method for multi-crane joint operation based on a multi-agent approach. Memarzadeh et al.^28^ and Park et al.^29^ realized the recognition, detection, and early warning of space conflict between pieces of construction equipment and workers using video streaming technology. Cheng et al.^30^ realized the remote virtual display of construction resources based on visualization technology and the identification of security risks with the real-time tracking of resource location data in construction scenarios. Wu et al.^31^ established a conflict monitoring and early warning method of cable operation in an arch dam based on GPS and GIS. Rybakova et al.^32^ proposed some algorithms and applied tools to improve the efficiency of design for accelerating construction. Dakwale et al.^33^ assessed energy efficiency by confirming various parameters including the regulatory and voluntary policies.

The above research focused on the safety issues in the pouring process of the dam block and on the interference behaviors between cable lifting and other construction processes, including conflict classification, conflict identification, and the quantitative representation of the spatial conflict. The research on construction efficiency loss is mainly focused on the efficiency loss caused by the quality of construction personnel, construction technology, the construction machinery configuration, the construction environment, and other factors. These research studies have produced some achievements in the evaluation, prediction, early warning, and optimization of the efficiency loss index. These research methods and ideas provide a certain foundation for carrying out more detailed and comprehensive research on the spatial–temporal conflict of construction machineries in a pouring block.

With the development of computer and system simulation technology, system simulation technology has become an important tool to solve complex discrete and random problem^34–40^, which provides the possibility for the simulation of the construction system^41,42^, especially for the construction process simulation of concrete arch dam with construction particularity and many limitations, including a complex construction environment, complex construction process, and cross-construction operation^7,43,44^. Guan et al.^45^ proposed a simulation method for high arch dam construction based on fuzzy Bayesian updating algorithm, which adopts the fuzzy set theory to process the original data by dynamical collecting the field construction parameters. The results showed that the accuracy of construction simulation parameters and simulation results is improved. Zhong et al.^46^ proposed a new method of construction scheme optimization for high arch dams based on stochastic dominance degrees and realized the optimization of construction schemes for high arch dams features with randomness in construction progress indexes. Song et al.^3^ presented a real-time construction simulation method coupling a concrete temperature field interval prediction model to solve the construction schedule deviation caused by simplified simulation model. Ren et al.^47^ proposed a visual simulation method of the pouring construction in pouring blocks for high arch dams based on Web augmented reality, and constructed a refined simulation model of pouring construction. These researches realized the cross-platform interactive analysis of Web browser. Li et al.^48^ established a set of temperature-stress coupling simulation system to be close to the actual engineering state and better for studying the working status of high arch dams, through optimizing the key thermodynamic parameters used in the simulation calculation.

In summary, the advantage of developing a simulation is that it more appropriately represents the dynamics of construction processes since it considers randomness and variability in the results^49,50^. In summary, the current researches of the construction simulation technology for hydraulic engineering focus on the analysis of the overall risk^51,52^, construction schedule^3,46,53^ and structural calculation^9,11,54,55^. There are few studies on the pouring construction system of blocks, and the simulation and virtual analysis of the pouring construction system considering the spatial–temporal conflict between the construction machinery are especially rare.

To this end, on the basis of the previous research for the quantitative calculation of the spatial–temporal conflict in pouring blocks, this paper presents the simulation objectives and modeling assumptions of the pouring construction system considering the spatial–temporal conflict, and constructs the system simulation model. Further, taking the pouring construction system of the typical blocks of the Baihetan dam as the simulation analysis object, the visualization simulation system of the spatial–temporal conflict is developed, which achieves information integration such as the quantification of the spatial–temporal conflict, the analysis of the influence effects, and the visualization of conflict information. The risk rate of spatial–temporal conflict in the whole construction process is simulated and calculated, and the outbreak condition of spatial–temporal conflict in the pouring process is simulated and rehearsed. The above research results can provide reference for the construction organization and management, scheme decision for construction safety and efficiency control, and important guidance for the fine and scientific construction of the pouring blocks in arch dams.

## Quantitative algorithm of spatial–temporal conflict of construction machinery

On the basis of previous research, according to the degree of spatial–temporal conflict of construction machinery and its influence on safety and efficiency, the types of spatial–temporal conflict are defined as efficiency loss, safety risk and physical collision, and the outer space of construction machinery is defined as physical space, safety space and efficiency space from inside to outside^7^.

The concept of a conflict bubble is introduced to describe a physical collision accident, security risk, and efficiency loss in a specific scenario and to lay the foundation for quantifying the physical collision accident rate, security risk rate, and efficiency loss rate of construction machinery^7^.

According to divide the machinery and its layered space into microelement units and divide the running trajectories of the construction machinery into the time-by-step trajectories of microelement units based on the temporal and spatial steps. Defining the mapping relationship between the change of the virtual space and the running time of entities during the running process as the time–space microelement (TSM). In the process, the complex and changeable volume units of the conflict bubble are discretized into the time-by-step micro-units, and the numbers of coincident micro-units and related algorithms are used to achieve the purpose of quantifying the physical collision accident rate, security risk rate, and efficiency loss rate^7^.

**Fig. 1:**
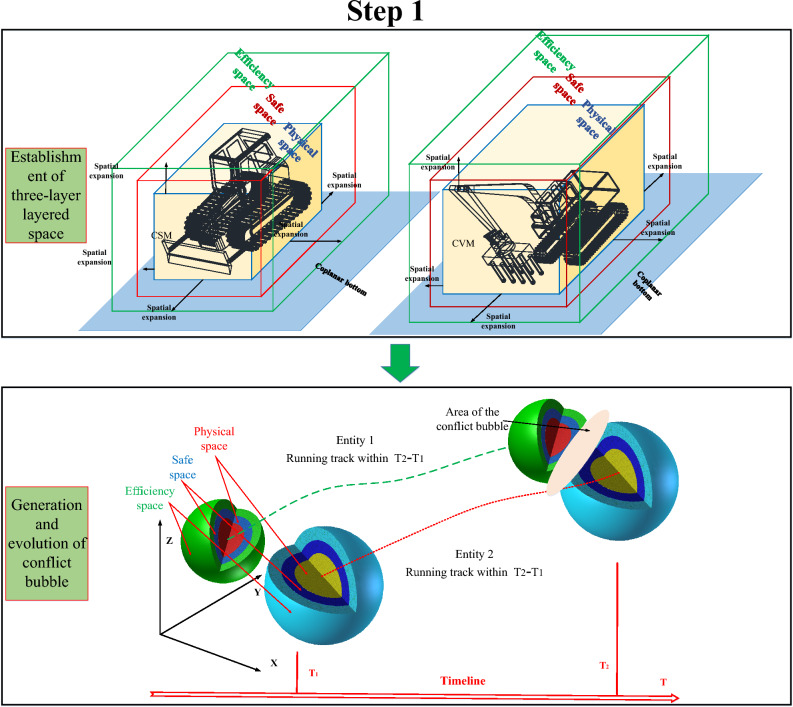
Step 1 of the quantization algorithm of the spatial–temporal conflict^7^.

**Fig. 2:**
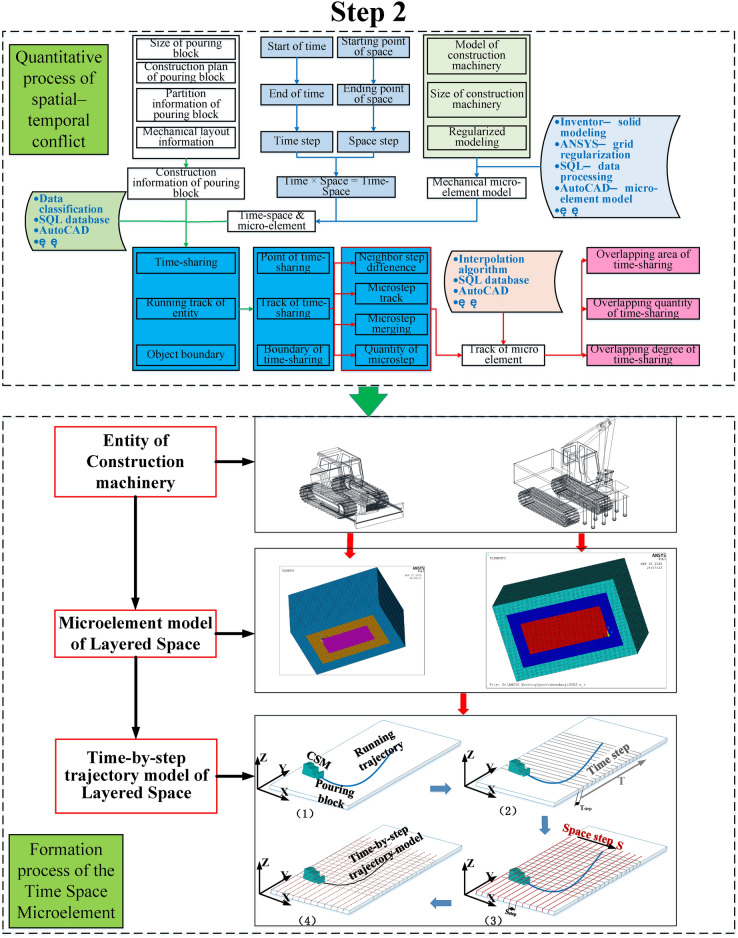
Step 2 of the quantization algorithm of the spatial–temporal conflict^7^.

**Fig. 3:**
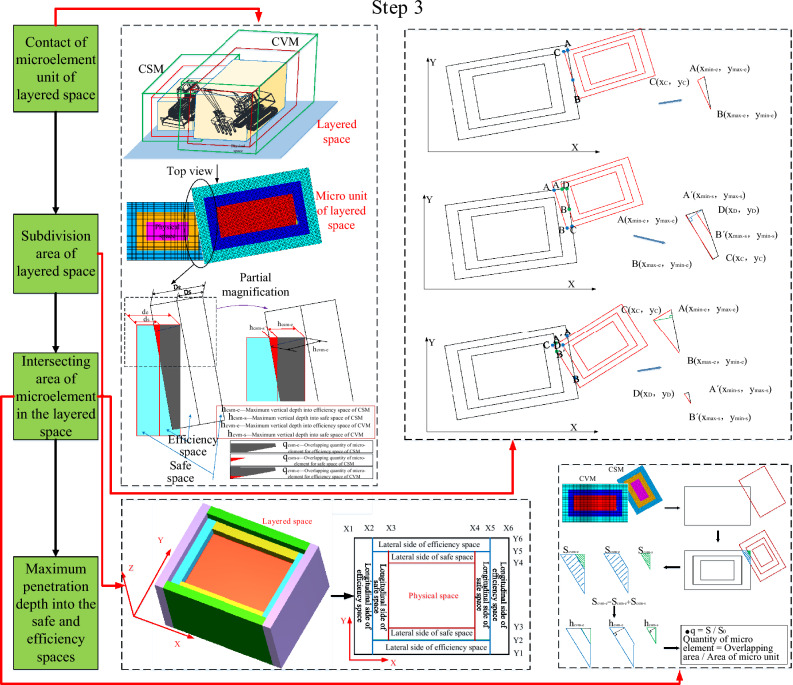
Step 3 of the quantization algorithm of the spatial–temporal conflict^7^.

**Fig. 4:**
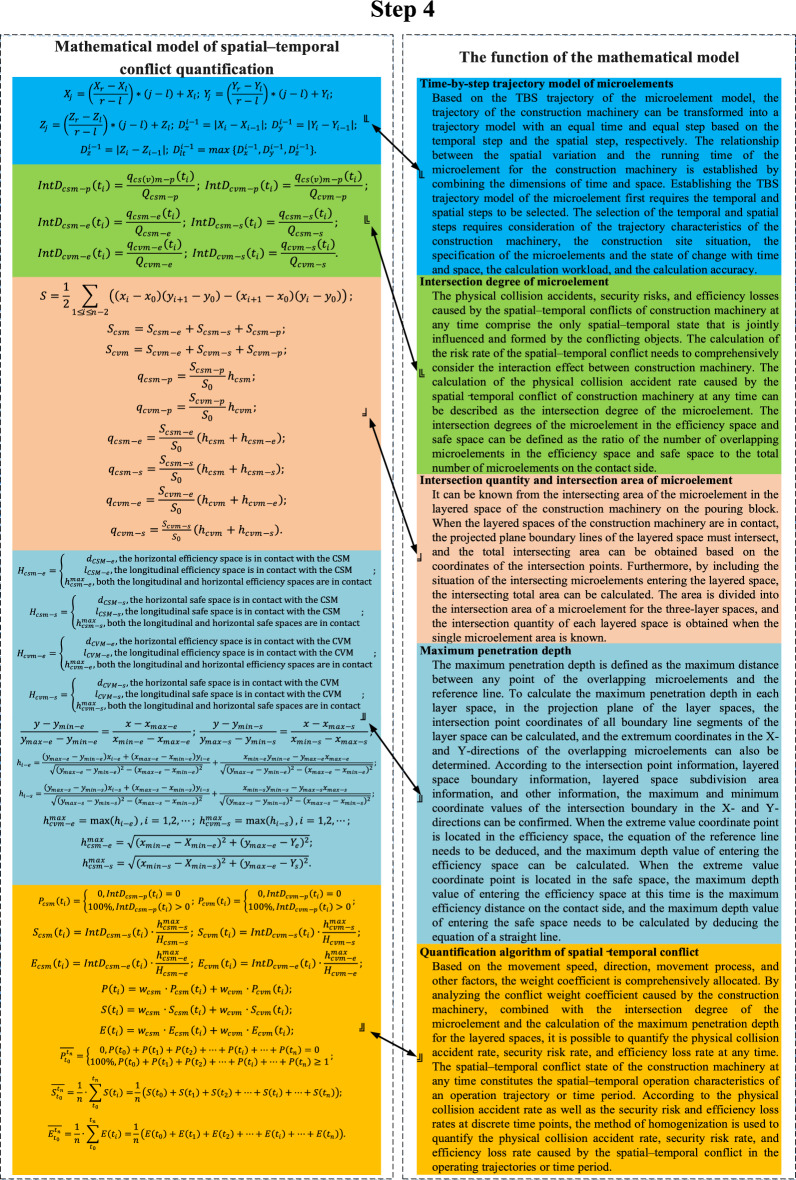
Mathematical model of spatial–temporal conflict quantification^7^.

Furthermore, the quantitative process of conflict bubble for construction machinery based on TSM is proposed. The introduction of the concept of a conflict bubble provides support for the vivid description of the safety risks and efficiency losses caused by the spatial–temporal conflict of construction machinery, which makes the safety risk and efficiency loss caused by the complex dynamic construction process more specific. Then, the safety risk and efficiency loss of construction machinery can be transformed into the safe space and efficiency space contact condition under the spatial–temporal conflict. The concept of the TSM is further introduced to discretize the complex and dynamically changing conflict bubble volume unit into time-by-step microelements, and the risk rate of spatial–temporal conflicts is quantified by using the intersection microelement quantity of the conflict bubble, intersection degree, and maximum penetration depth. the quantification algorithm of the spatial–temporal conflict between construction machinery in the dynamic running process were proposed based on the TSM, which can quantify the physical collision accident rate, security risk rate, and efficiency loss rate of construction machinery at any time point or time period. Based on these studies, the quantitative algorithm of spatial–temporal conflict of construction machinery can be divided into four steps^7^, as shown in Fig 1,2,3,4.

Since the previous research has conducted an in-depth study on the quantitative algorithm of spatial–temporal conflict of construction machinery, the quantitative algorithm of spatial–temporal conflict is not described in detail. This chapter provides a theoretical basis for the early warning and alarm of the safety risk rate and efficiency loss rate of construction machinery by clarifying the quantitative algorithm architecture of spatial–temporal conflict and combing the quantitative process of spatial–temporal conflict. It provides a theoretical basis for further development of the simulation of the pouring construction system of the block considering the spatial–temporal conflict.

A certain trajectory of the construction machinery is set to be in a constant speed state, and this trajectory is divided into $m$ segments of equal time length, which include $\left(m+1\right)$ nodes. Assuming that the running time of this trajectory is $T$, then $m=T/{T}_{step}$. The running trajectory of the $\left(m+1\right)$ nodes divided by the same time length are numbered successively, and their coordinates are $\left({X}_{i},{Y}_{i},{Z}_{i}\right)$, where $i$ is the coordinate point number, and $0<i\le m+1$. These nodes are defined as trajectory points, the known points that reflect the characteristics of the construction trajectory of the storehouse surface are defined as key trajectory points, and the remaining trajectory points are non-key trajectory points. A non-critical trajectory point $j$ point ($0<j\le i$) is defined to be located in the key point interval $\left[l,r\right]$ ($0<l\le j\le r$). The coordinates of non-critical trajectory points $\left({X}_{j},{Y}_{j},{Z}_{j}\right)$are defined. Based on the temporal step, the trajectory of the construction machinery is divided into continuous straight-line segment trajectory points, and the spatial state change process is also divided. Then, according to the spatial step ${S}_{step}$, the trajectory between adjacent trajectory points is divided again, and the regular microelement time-sharing step-by-step trajectory is established. First, according to the coordinates of adjacent trajectory points $\left(i-1\right)$ and $i$, the neighbor step differences ${D}_{x}^{i-1}, {D}_{y}^{i-1}$, and ${D}_{z}^{i-1}$ between adjacent trajectory points in the $\left(i-1\right)$ period can be calculated, that is, the distance between adjacent trajectory points is the projected size in the $X$-, $Y$-, and $Z$-directions, and then the numbers of micro-steps in the $X$-, $Y$-, and $Z$-directions are calculated based on the neighbor step difference and the spatial step size. Here, the maximum neighbor step difference ${D}_{lt}^{i-1}$ of adjacent trajectory points is introduced, and $\left\lfloor {D_{lt}^{i - 1} /S_{step} } \right\rfloor$ (rounded down) is taken as the number of micro-steps. The number of micro-steps is the size of the distance projection size between adjacent trajectory points in the $X$-, $Y$-, and $Z$-directions based on the spatial step^7^.

With the contact of the layered space between the CSM and CVM as an example, the calculation of the physical collision accident rate can be described as the intersection degree of the microelement ${IntD}_{cs\left(v\right)m-p}\left({t}_{i}\right)$ for the physical space. The intersection quantity of the microelement in the physical space between the CSM and CVM at any time ${t}_{i}$ is defined as ${q}_{cs\left(v\right)m-p}\left({t}_{i}\right)$. At any time ${t}_{i}$, the intersection degrees of the microelement in the efficiency space and safe space for the CSM, ${IntD}_{csm-e}\left({t}_{i}\right)$ and ${IntD}_{cvm-e}\left({t}_{i}\right)$, respectively, and for the CVM, ${IntD}_{csm-s}\left({t}_{i}\right)$, ${IntD}_{cvm-s}\left({t}_{i}\right)$. Where ${q}_{cs\left(v\right)m-p}\left({t}_{i}\right)$, ${q}_{csm-e}\left({t}_{i}\right)$, ${q}_{csm-s}\left({t}_{i}\right)$, ${q}_{cvm-e}\left({t}_{i}\right)$, and ${q}_{cvm-s}\left({t}_{i}\right)$ are the number of overlapping microelements in the physical space for the CSM and CVM, the number of overlapping microelements in the efficiency space for the CSM, the number of overlapping microelements in the safe space for the CSM, the number of overlapping microelements in the efficiency space for the CVM, and the number of overlapping microelements in the safe space for the CVM, respectively. ${Q}_{csm-p}$ and ${Q}_{cvm-p}$ are the number of microelements on the contact side of the physical space for the CSM and CVM, respectively, ${Q}_{csm-e}$ and ${Q}_{cvm-e}$ are the number of microelements on the contact side of the efficiency space for the CSM and CVM, respectively, and ${Q}_{csm-s}$ and ${Q}_{cvm-s}$ are the number of microelements on the contact side of the safe space for the CSM and CVM, respectively^7^.

The intersection points in the clockwise or counterclockwise directions are set as $({x}_{0}, {y}_{0}), ({x}_{1}, {y}_{1}),\left({x}_{2}, {y}_{2}\right), \cdots \cdots , ({x}_{n-1}, {y}_{n-1}).$ The intersection area in the intersecting projected plane is $S$. Where ${S}_{csm-e}$, ${S}_{csm-s}$, and ${S}_{csm-p}$ are the intersection areas of the efficiency, safe, and physical space for the CSM, respectively, and ${S}_{cvm-e}$, ${S}_{cvm-s}$, and ${S}_{cvm-p}$ are the intersection areas of the efficiency, safe, and physical space s for the CVM, respectively. Where ${q}_{csm-p}$, ${q}_{csm-s},$ and ${q}_{csm-e}$ are the intersection quantities of the physics, safe, and efficiency spaces for the CSM, respectively, ${q}_{cvm-p}$, ${q}_{cvm-s}$, and ${q}_{cvm-e}$ are the intersection quantities of the physics, safe, and efficiency spaces for the CVM, respectively, and ${S}_{0}$ is the single microelement area^7^.

When the layered space is in contact, the maximum penetration depths in the efficiency space and the safe space of the CSM are defined as ${h}_{csm-e}^{max}$ and ${h}_{csm-s}^{max}$, respectively, and the maximum penetration depths in the efficiency space and the safe space of the CVM are defined as ${h}_{cvm-e}^{max}$ and ${h}_{cvm-s}^{max}$, respectively. The depths of the contact side efficiency space and safety space of the CSM are defined as ${H}_{csm-e}$ and ${H}_{csm-s}$, respectively, and the depths of the contact side efficiency space and safety space of the CVM are defined as ${H}_{cvm-e}$ and ${H}_{cv\text{m}-s}$, respectively. The X- and Y-axis extreme coordinates of the overlapping microelements in the efficiency space and safe space projection planes are ${x}_{max-e}$, ${x}_{min-e}$, ${y}_{max-e}$, ${y}_{min-e}$, and ${x}_{max-s}$, ${x}_{min-s}$, ${y}_{max-s}, {y}_{min-s}$, respectively. The extreme value coordinates entering the efficiency and safe spaces are defined as $({X}_{min-e},{Y}_{e})$ and $({X}_{min-s},{Y}_{s})$, respectively. Where ${h}_{i-e}$ and ${h}_{i-s}$ are the depth values of entering the efficiency space and the safe space, respectively. Where ${h}_{cvm-e}^{max}$ and ${h}_{cvm-s}^{max}$ are the maximum depth values of entering the efficiency space and the safe space for the CVM, where ${h}_{csm-e}^{max}$ and ${h}_{csm-s}^{max}$ are the maximum depth values for entering the efficiency space and the safe space for the CSM, respectively^7^.

By defining physical collision accident rates, security risk rates, and efficiency loss rates of the CSM, as ${P}_{csm}\left({t}_{i}\right)$, ${S}_{csm}\left({t}_{i}\right)$, and ${E}_{csm}\left({t}_{i}\right)$ respectively, and those of the CVM as ${P}_{cvm}\left({t}_{i}\right)$, ${S}_{cvm}\left({t}_{i}\right)$, and ${E}_{cvm}\left({t}_{i}\right)$. Where $i\in N$, and $P\left({t}_{i}\right), S\left({t}_{i}\right)$, and $E\left({t}_{i}\right)$ are the physical collision accident rate, security risk rate, and efficiency loss rate between the CSM and CVM at ${t}_{i}$ time, respectively. ${w}_{csm}$ and ${w}_{cvm}$ are the weight coefficients of the CSM and CVM, respectively, in the spatial–temporal conflict generated during the construction process. Where $n\in N$, and $\overline{{P}_{{t}_{0}}^{{t}_{n}}}, \overline{{S}_{{t}_{0}}^{{t}_{n}}}$, and $\overline{{E}_{{t}_{0}}^{{t}_{n}}}$ are the physical collision accident rate, security risk rate, and efficiency loss rate, respectively, between the CSM and CVM during the time period from ${t}_{0}$ to ${t}_{n}$^7^.

## Mathematical model for calculating the threshold of the spatial–temporal conflict risk rate

The mathematical model for calculating the threshold of safety risk rate and efficiency loss rate of space–time conflict of construction machinery is constructed, which can provide a theoretical basis for early warning and alarm of safety risk rate and efficiency loss rate of construction machinery.

To determine whether the spatial–temporal conflict of construction machinery will cause greater harm at a certain time, it is necessary to calculate the threshold of the spatial–temporal conflict risk rate to guide the construction organization and management, evaluate the spatial–temporal conflict, and adjust the construction measures on time. To achieve an early warning and alarm for physical collision accidents caused by spatial–temporal conflict during the pouring construction process, the moment of the first physical collision accident is defined as ${t}_{c}$. The security risk rate and the efficiency loss rate caused by the spatial–temporal conflict at this time, as determined by setting ${t}_{c}$ backward with a certain safety margin time, can be quantified.

Vehicles brake urgently during high-speed driving, and the safety distance threshold is determined by the three-second principle^56–58^. This rule is introduced into the construction machinery operation process in the pouring block. Since the construction machinery has low-speed and heavy-load operations, the CSM and the CVM both have low running speeds, fast braking operations, and short reaction times. The emergency response time of normal personnel is about 1.4–1.6 s. According to this, the moment when the first physical collision accident between the CSM and the CVM occurs is defined as ${t}_{c}$. Setting ${t}_{c}$ backward by 2 s, the security risk rate and the efficiency loss rate caused by the spatial–temporal conflict can be quantified. Then, the corresponding quantified values can be defined as the threshold of the security risk rate and efficiency loss rate.

**Fig. 5:**
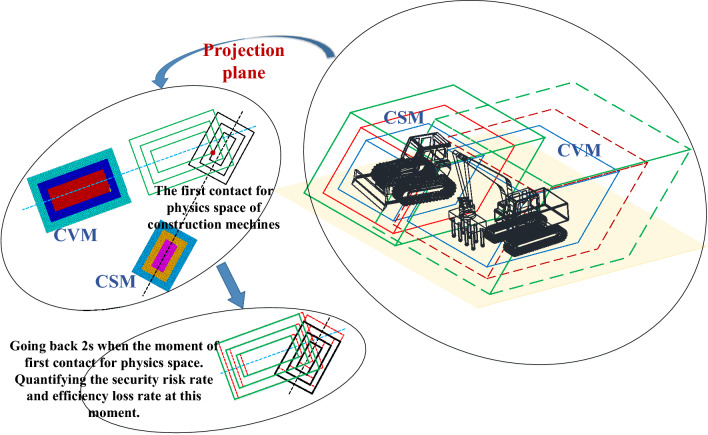
Schematic diagram of threshold determination of security risk rate and efficiency loss rate.

Figure 5 shows a schematic diagram of the threshold determination of the security risk rate and efficiency loss rate.

**Fig. 6:**
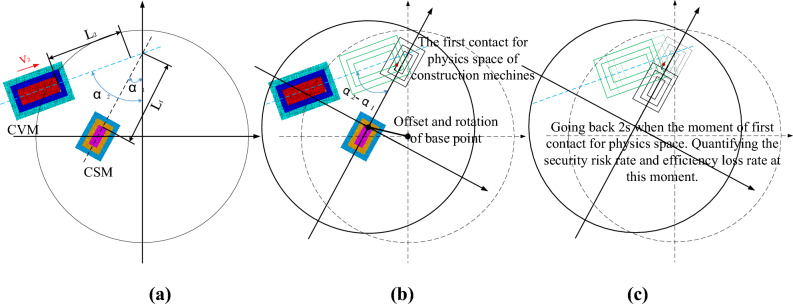
Schematic diagram of the threshold calculation model of security risk rate and efficiency loss rate. (**a**) The initial state of CSM and CVM. (**b**) The first contact for physics space of construction machines. (**c**) Going back 2 s when the moment of first contact for physics space. Quantifying the security risk rate and efficiency loss rate at this moment.

The CSM and CVM run in a certain operating state, as shown in Fig. 6a. In the projected plane reference coordinate system, the CSM runs at a speed ${v}_{1}$ and a positive angle ${\alpha }_{1}$ along the Y-axis, and the CVM runs at a speed ${v}_{2}$ and a positive angle ${\alpha }_{2}$ along the Y-axis. When the time runs to a certain moment ${t}_{c}$, the CSM and the CVM have the first contact in the physical space, as shown in Fig. 6b. Going back 2 s when the moment of first contact for physics space. Quantifying the security risk rate and efficiency loss rate at this moment, as shown in Fig. 6c.

**Fig. 7:**
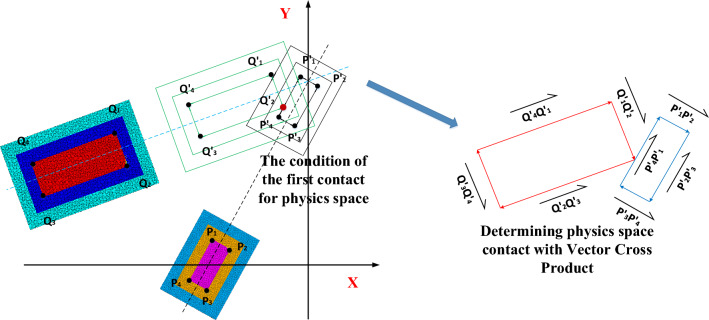
Schematic diagram of physical space contact determination model based on the vector cross-product.

At the initial moment, the four vertices of the physics space in the projected plane are points $${P}_{1}$$, $${P}_{2}$$, $${P}_{3}$$, and $${P}_{4}$$ for the CSM and $${Q}_{1}$$, $${Q}_{2}$$, $${Q}_{3}$$, and $${Q}_{4}$$ for the CVM, whose coordinates are $$({x}_{1},{y}_{1})$$, $$\left({x}_{2},{y}_{2}\right)$$, $$\left({x}_{3},{y}_{3}\right)$$, and $$({x}_{4},{y}_{4})$$ and $$({x}^{\prime}_{1},{y}^{\prime}_{1})$$, $$({x}^{\prime}_{2},{y}^{\prime}_{2})$$, $$({x}^{\prime}_{3},{y}^{\prime}_{3})$$, and $$({x}^{\prime}_{4},{y}^{\prime}_{4})$$, respectively. When the CSM and the CVM move the respective distances of $${L}_{1}$$ and $${L}_{2}$$ simultaneously to achieve their first contact in the physics space, the four vertices of the CSM shift from $${P}_{1}$$, $${P}_{2}$$, $${P}_{3}$$ and $${P}_{4}$$ to $${P}^{\prime}_{1}({x}_{1}+{v}_{1}{t}_{c}sin{\alpha }_{1},{y}_{1}\,$$ $$+{v}_{1}{t}_{c}cos{\alpha }_{1})$$, $${P}^{\prime}_{2}\left({x}_{2}+{v}_{1}{t}_{c}sin{\alpha}_{1},{y}_{2}+{v}_{1}{t}_{c}cos{\alpha }_{1}\right)$$, $${P}^{\prime}_{3}\left({x}_{3}+{v}_{1}{t}_{c}sin{\alpha}_{1},{y}_{3}{+v}_{1}{t}_{c}cos{\alpha}_{1}\right)$$, and $${P}^{\prime}_{4}({x}_{4}+{v}_{1}{t}_{c}sin{\alpha }_{1},{y}_{4}$$$$+{v}_{1}{t}_{c}cos{\alpha}_{1})$$ respectively, and the four vertices of the CVM shift from $${Q}_{1}$$ $${Q}_2$$, $${Q}_{3}$$, $${Q}_{4}$$ to $${Q}^{\prime}_{1}({x}^{\prime}_{1}+{v}_{2}{t}_{c}sin{\alpha}_{2},{y}^{\prime}_{1}$$ $$+{v}_{2}{t}_{c}cos{\alpha }_{2})$$, $${Q}^{\prime}_{2}({x}^{\prime}_{2}+{v}_{2}{t}_{c}sin{\alpha }_{2},\,{y}^{\prime}_{2}$$ $$+{v}_{2}{t}_{c}cos{\alpha }_{2})$$, and $${Q}^{\prime}_{4}({x}^{\prime}_{4}+{v}_{2}{t}_{c}sin{\alpha }_{2},{y}^{\prime}_{4}$$ $$+{v}_{2}{t}_{c}cos{\alpha }_{2})$$ respectively At this moment, there is an intersection between the four line segments of the physical space boundary. According to the vector intersection determination criterion, the basic vector is constructed based on the four vertices of the projection plane of the physics space, and the vector cross-product is used to determine whether there is contact. Figure 7 shows the schematic diagram of the determination model of physical space contact.

When the physics space has the first contact between the CSM and the CVM, there existed as shown in Eq. (1):

1 $$\begin{gathered} \left\{ {\overset{\lower0.5em\hbox{$\smash{\scriptscriptstyle\rightharpoonup}$}}{{P_{1} ^{\prime}P_{2} ^{\prime}}} \times \left\{ {\overset{\lower0.5em\hbox{$\smash{\scriptscriptstyle\rightharpoonup}$}}{{Q_{1} ^{\prime}Q_{2} ^{\prime}}} ,\overset{\lower0.5em\hbox{$\smash{\scriptscriptstyle\rightharpoonup}$}}{{Q_{2} ^{\prime}Q_{3} ^{\prime}}} ,\overset{\lower0.5em\hbox{$\smash{\scriptscriptstyle\rightharpoonup}$}}{{Q_{3} ^{\prime}Q_{4} ^{\prime}}} ,\overset{\lower0.5em\hbox{$\smash{\scriptscriptstyle\rightharpoonup}$}}{{Q_{4} ^{\prime}Q_{1} ^{\prime}}} } \right\}} \right\} \cup \left\{ {\overset{\lower0.5em\hbox{$\smash{\scriptscriptstyle\rightharpoonup}$}}{{P_{2} ^{\prime}P_{3} ^{\prime}}} \times \left\{ {\overset{\lower0.5em\hbox{$\smash{\scriptscriptstyle\rightharpoonup}$}}{{Q_{1} ^{\prime}Q_{2} ^{\prime}}} ,\overset{\lower0.5em\hbox{$\smash{\scriptscriptstyle\rightharpoonup}$}}{{Q_{2} ^{\prime}Q_{3} ^{\prime}}} ,\overset{\lower0.5em\hbox{$\smash{\scriptscriptstyle\rightharpoonup}$}}{{Q_{3} ^{\prime}Q_{4} ^{\prime}}} ,\overset{\lower0.5em\hbox{$\smash{\scriptscriptstyle\rightharpoonup}$}}{{Q_{4} ^{\prime}Q_{1} ^{\prime}}} } \right\}} \right\} \hfill \\ \cup \left\{ {\overset{\lower0.5em\hbox{$\smash{\scriptscriptstyle\rightharpoonup}$}}{{P_{3} ^{\prime}P_{4} ^{\prime}}} \times \left\{ {\overset{\lower0.5em\hbox{$\smash{\scriptscriptstyle\rightharpoonup}$}}{{Q_{1} ^{\prime}Q_{2} ^{\prime}}} ,\overset{\lower0.5em\hbox{$\smash{\scriptscriptstyle\rightharpoonup}$}}{{Q_{2} ^{\prime}Q_{3} ^{\prime}}} ,\overset{\lower0.5em\hbox{$\smash{\scriptscriptstyle\rightharpoonup}$}}{{Q_{3} ^{\prime}Q_{4} ^{\prime}}} ,\overset{\lower0.5em\hbox{$\smash{\scriptscriptstyle\rightharpoonup}$}}{{Q_{4} ^{\prime}Q_{1} ^{\prime}}} } \right\}} \right\} \cup \left\{ {\overset{\lower0.5em\hbox{$\smash{\scriptscriptstyle\rightharpoonup}$}}{{P_{4} ^{\prime}P_{1} ^{\prime}}} \times \left\{ {\overset{\lower0.5em\hbox{$\smash{\scriptscriptstyle\rightharpoonup}$}}{{Q_{1} ^{\prime}Q_{2} ^{\prime}}} ,\overset{\lower0.5em\hbox{$\smash{\scriptscriptstyle\rightharpoonup}$}}{{Q_{2} ^{\prime}Q_{3} ^{\prime}}} ,\overset{\lower0.5em\hbox{$\smash{\scriptscriptstyle\rightharpoonup}$}}{{Q_{3} ^{\prime}Q_{4} ^{\prime}}} ,\overset{\lower0.5em\hbox{$\smash{\scriptscriptstyle\rightharpoonup}$}}{{Q_{4} ^{\prime}Q_{1} ^{\prime}}} } \right\}} \right\} \hfill \\ \ne \overset{\lower0.5em\hbox{$\smash{\scriptscriptstyle\rightharpoonup}$}}{0} ,\;\;\;\;\;\;\;\;\left| {\alpha_{2} - \alpha_{1} } \right| \hfill \\ \ne 0^\circ \;\;\;\;or\;\;\;\;180^\circ \;\;\;\;or\;\;\;\;360^\circ \hfill \\ \end{gathered}$$

When the physics space has the first contact between the CSM and the CVM, there existed as shown in Eq. (2):

2 $$\begin{gathered} \left\{ {\overset{\lower0.5em\hbox{$\smash{\scriptscriptstyle\rightharpoonup}$}}{{P_{1} ^{\prime}P_{2} ^{\prime}}} \times \left\{ {\overset{\lower0.5em\hbox{$\smash{\scriptscriptstyle\rightharpoonup}$}}{{Q_{1} ^{\prime}Q_{2} ^{\prime}}} ,\overset{\lower0.5em\hbox{$\smash{\scriptscriptstyle\rightharpoonup}$}}{{Q_{2} ^{\prime}Q_{3} ^{\prime}}} ,\overset{\lower0.5em\hbox{$\smash{\scriptscriptstyle\rightharpoonup}$}}{{Q_{3} ^{\prime}Q_{4} ^{\prime}}} ,\overset{\lower0.5em\hbox{$\smash{\scriptscriptstyle\rightharpoonup}$}}{{Q_{4} ^{\prime}Q_{1} ^{\prime}}} } \right\}} \right\} \cup \left\{ {\overset{\lower0.5em\hbox{$\smash{\scriptscriptstyle\rightharpoonup}$}}{{P_{2} ^{\prime}P_{3} ^{\prime}}} \times \left\{ {\overset{\lower0.5em\hbox{$\smash{\scriptscriptstyle\rightharpoonup}$}}{{Q_{1} ^{\prime}Q_{2} ^{\prime}}} ,\overset{\lower0.5em\hbox{$\smash{\scriptscriptstyle\rightharpoonup}$}}{{Q_{2} ^{\prime}Q_{3} ^{\prime}}} ,\overset{\lower0.5em\hbox{$\smash{\scriptscriptstyle\rightharpoonup}$}}{{Q_{3} ^{\prime}Q_{4} ^{\prime}}} ,\overset{\lower0.5em\hbox{$\smash{\scriptscriptstyle\rightharpoonup}$}}{{Q_{4} ^{\prime}Q_{1} ^{\prime}}} } \right\}} \right\} \hfill \\ \cup \left\{ {\overset{\lower0.5em\hbox{$\smash{\scriptscriptstyle\rightharpoonup}$}}{{P_{3} ^{\prime}P_{4} ^{\prime}}} \times \left\{ {\overset{\lower0.5em\hbox{$\smash{\scriptscriptstyle\rightharpoonup}$}}{{Q_{1} ^{\prime}Q_{2} ^{\prime}}} ,\overset{\lower0.5em\hbox{$\smash{\scriptscriptstyle\rightharpoonup}$}}{{Q_{2} ^{\prime}Q_{3} ^{\prime}}} ,\overset{\lower0.5em\hbox{$\smash{\scriptscriptstyle\rightharpoonup}$}}{{Q_{3} ^{\prime}Q_{4} ^{\prime}}} ,\overset{\lower0.5em\hbox{$\smash{\scriptscriptstyle\rightharpoonup}$}}{{Q_{4} ^{\prime}Q_{1} ^{\prime}}} } \right\}} \right\} \cup \left\{ {\overset{\lower0.5em\hbox{$\smash{\scriptscriptstyle\rightharpoonup}$}}{{P_{4} ^{\prime}P_{1} ^{\prime}}} \times \left\{ {\overset{\lower0.5em\hbox{$\smash{\scriptscriptstyle\rightharpoonup}$}}{{Q_{1} ^{\prime}Q_{2} ^{\prime}}} ,\overset{\lower0.5em\hbox{$\smash{\scriptscriptstyle\rightharpoonup}$}}{{Q_{2} ^{\prime}Q_{3} ^{\prime}}} ,\overset{\lower0.5em\hbox{$\smash{\scriptscriptstyle\rightharpoonup}$}}{{Q_{3} ^{\prime}Q_{4} ^{\prime}}} ,\overset{\lower0.5em\hbox{$\smash{\scriptscriptstyle\rightharpoonup}$}}{{Q_{4} ^{\prime}Q_{1} ^{\prime}}} } \right\}} \right\} \hfill \\ = \overset{\lower0.5em\hbox{$\smash{\scriptscriptstyle\rightharpoonup}$}}{0} ,\,\;\;{\text{and}}\;\;\;\left\{ {\overset{\lower0.5em\hbox{$\smash{\scriptscriptstyle\rightharpoonup}$}}{{P_{1} ^{\prime}P_{2} ^{\prime}}} \times \left\{ {\overset{\lower0.5em\hbox{$\smash{\scriptscriptstyle\rightharpoonup}$}}{{P_{1} ^{\prime}Q_{1} ^{\prime}}} ,\overset{\lower0.5em\hbox{$\smash{\scriptscriptstyle\rightharpoonup}$}}{{P_{1} ^{\prime}Q_{2} ^{\prime}}} ,\overset{\lower0.5em\hbox{$\smash{\scriptscriptstyle\rightharpoonup}$}}{{P_{1} ^{\prime}Q_{3} ^{\prime}}} ,\overset{\lower0.5em\hbox{$\smash{\scriptscriptstyle\rightharpoonup}$}}{{P_{1} ^{\prime}Q_{4} ^{\prime}}} } \right\}} \right\} \cup \left\{ {\overset{\lower0.5em\hbox{$\smash{\scriptscriptstyle\rightharpoonup}$}}{{P_{2} ^{\prime}P_{3} ^{\prime}}} \times \left\{ {\overset{\lower0.5em\hbox{$\smash{\scriptscriptstyle\rightharpoonup}$}}{{P_{2} ^{\prime}Q_{1} ^{\prime}}} ,\overset{\lower0.5em\hbox{$\smash{\scriptscriptstyle\rightharpoonup}$}}{{P_{2} ^{\prime}Q_{2} ^{\prime}}} ,\overset{\lower0.5em\hbox{$\smash{\scriptscriptstyle\rightharpoonup}$}}{{P_{2} ^{\prime}Q_{3} ^{\prime}}} ,\overset{\lower0.5em\hbox{$\smash{\scriptscriptstyle\rightharpoonup}$}}{{P_{2} ^{\prime}Q_{4} ^{\prime}}} } \right\}} \right\} \hfill \\ \cup \left\{ {\overset{\lower0.5em\hbox{$\smash{\scriptscriptstyle\rightharpoonup}$}}{{P_{3} ^{\prime}P_{4} ^{\prime}}} \times \left\{ {\overset{\lower0.5em\hbox{$\smash{\scriptscriptstyle\rightharpoonup}$}}{{P_{3} ^{\prime}Q_{1} ^{\prime}}} ,\overset{\lower0.5em\hbox{$\smash{\scriptscriptstyle\rightharpoonup}$}}{{P_{3} ^{\prime}Q_{2} ^{\prime}}} ,\overset{\lower0.5em\hbox{$\smash{\scriptscriptstyle\rightharpoonup}$}}{{P_{3} ^{\prime}Q_{3} ^{\prime}}} ,\overset{\lower0.5em\hbox{$\smash{\scriptscriptstyle\rightharpoonup}$}}{{P_{3} ^{\prime}Q_{4} ^{\prime}}} } \right\}} \right\} \cup \left\{ {\overset{\lower0.5em\hbox{$\smash{\scriptscriptstyle\rightharpoonup}$}}{{P_{4} ^{\prime}P_{1} ^{\prime}}} \times \left\{ {\overset{\lower0.5em\hbox{$\smash{\scriptscriptstyle\rightharpoonup}$}}{{P_{4} ^{\prime}Q_{1} ^{\prime}}} ,\overset{\lower0.5em\hbox{$\smash{\scriptscriptstyle\rightharpoonup}$}}{{P_{4} ^{\prime}Q_{2} ^{\prime}}} ,\overset{\lower0.5em\hbox{$\smash{\scriptscriptstyle\rightharpoonup}$}}{{P_{4} ^{\prime}Q_{3} ^{\prime}}} ,\overset{\lower0.5em\hbox{$\smash{\scriptscriptstyle\rightharpoonup}$}}{{P_{4} ^{\prime}Q_{4} ^{\prime}}} } \right\}} \right\} = \overset{\lower0.5em\hbox{$\smash{\scriptscriptstyle\rightharpoonup}$}}{0} , \hfill \\ \left| {\alpha_{2} - \alpha_{1} } \right| = 0^\circ \,{\text{or}}\,\,{180}^\circ \,\,{\text{or}}\,\,{360}^\circ \hfill \\ \end{gathered}$$

Finally, according to the movement of the spatial coordinates at time $({t}_{c}-2)$, the security risk rate and the efficiency loss rate in the current state can be quantified to obtain the quantification thresholds of the security risk rate and the efficiency loss rate of the spatial–temporal conflict in the current operating state.

## Simulation of the pouring construction system considering mechanical spatial–temporal conflict

The unique construction characteristics and composition of the pouring construction system form a relatively mature construction process and a dynamically adjusted on-site construction organization mode. In the process of pouring construction organization design and actual construction, there are some limitations in the on-site construction management mode based on traditional two-dimensional construction drawings, such as structural conflicts caused by spatial dimension deviation during actual construction and construction conflicts caused by insufficient construction organization coordination. The occurrence of these conflicts or interference is due to the relative lack of consideration of space resources in the traditional two-dimensional design process and the lack of systematic and comprehensive virtual simulation analysis of the construction process. For this reason, the cyclic process of the construction machinery during the pouring operation is conducted based on the analysis of the spatial–temporal conflict system to understand the outbreak stage of the spatial–temporal conflict of construction machinery. Moreover, the simulation goals and modeling assumptions of the construction machinery spatial–temporal conflict system are proposed, and the system simulation model of the pouring construction process considering the construction machinery spatial–temporal conflict is constructed.

### Operation cycle for construction machinery in pouring block

**Fig. 8:**
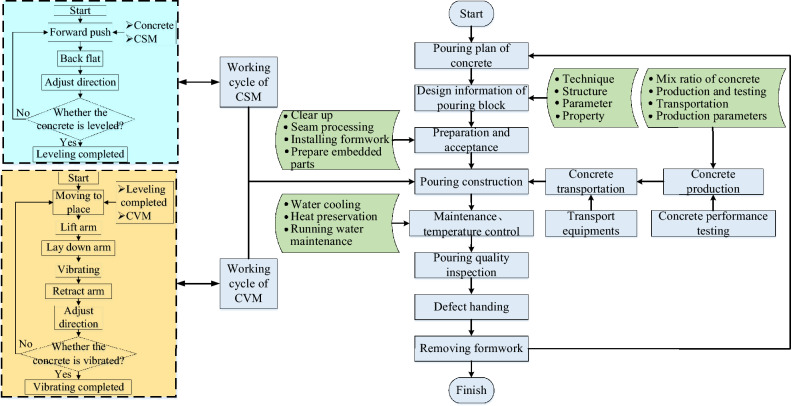
Construction process flowchart for the pouring block.

All of the construction system behaviors in the concrete arch dam are carried out around a pouring block, which is an important unit of an arch dam. The quality, safety, and progress of the construction process directly affect the dam pouring quality, construction safety, and economic benefits. To clarify the operation actions of each construction machine, the resources required for each step, and the cyclic process of construction machinery, the construction cycle model of the CSM and the CVM is constructed, which is integrated into the pouring construction process. Figure 8 shows the construction process flowchart for the pouring block.

### Target and principle of system simulation

#### Simulation target

**Fig. 9:**
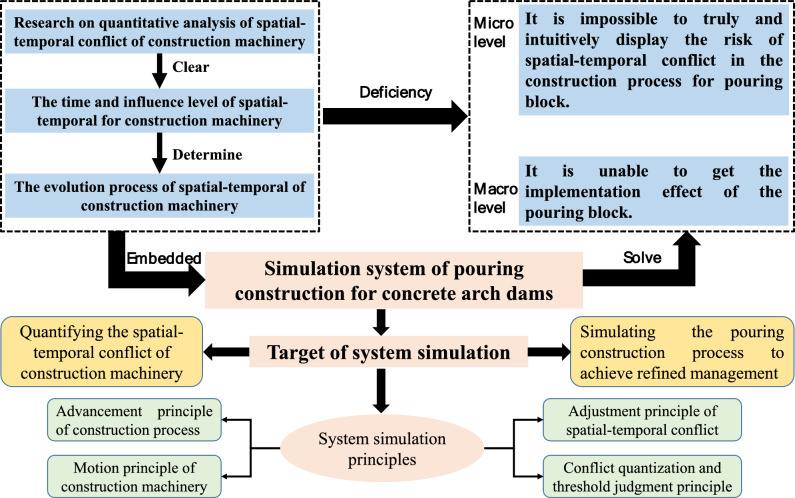
Schematic diagram of system simulation target and principle.

The spatial–temporal conflict of construction machinery for the pouring block of an arch dam is affected by many factors, such as the size of the pouring block, construction technology, machinery configuration, machinery running trajectory, and running speed. Many of the above factors are often uncertain and dynamic. Therefore, the conventional analytical mathematical model cannot be used to quantify the spatial–temporal conflict of the construction machinery for the pouring block. Computer simulation technology is an effective means to analyze and solve complex system problems. For the spatial–temporal conflict system of construction machinery, a mathematical model is established based on discrete system theory. Combining the constructed spatial–temporal conflict quantification method of construction machinery, the physical collision risk rate, security risk rate, and efficiency loss rate caused by the spatial–temporal conflict during the construction process can be quantified, and the simulation and analysis of the spatial–temporal conflict system of the construction machinery for the pouring block are realized. As shown in Fig. 9 is the schematic diagram of system simulation target and principle.

#### Simulation principle

1. The advancement principle of the construction process.
2. The motion principle of construction machinery.
3. The adjustment principle of spatial–temporal conflict.
4. The spatial–temporal conflict quantization and threshold judgment principle.

#### Modeling assumptions

In the development of the spatial–temporal conflict simulation model for construction machinery in the pouring block of arch dams, the following assumptions were formulated based on the simulation objectives:

1. The concrete production and transportation system is assumed to operate normally to meet the construction demands of the pouring block. The cable crane unloading process is treated as a discrete event with fixed time duration, predetermined discharge points, and constant discharge volume;
2. The construction in the pouring block is uniformly implemented using a zone- and strip-based methodology;
3. Each zone in the pouring block is equipped with one CSM, one CVM, and one cable crane. Auxiliary equipment is assumed to meet basic operational requirements;
4. The spreading process of the CSM is confined to the designated strip boundaries. The CVM initiates operations from an adjacent strip within the same zone and remains within the strip boundaries during routine compaction, except during the final compaction phase;
5. At the initial stage of strip construction, the CSM commences material distribution after two batches of concrete have been unloaded by the cable crane. The spreading process is strictly controlled to proceed sequentially from one transverse joint face to the opposite transverse joint face, with the CVM following an identical sequential operation pattern;
6. The layer thickness of concrete placements is assumed not to affect the planar orientation of the concrete spreading and vibrating machines, ensuring their working planes remain parallel to the pouring block at all times.

### Simulation model of the pouring construction system in the pouring block

#### Evolutionary model of spatial–temporal conflict of construction machinery

**Fig. 10:**
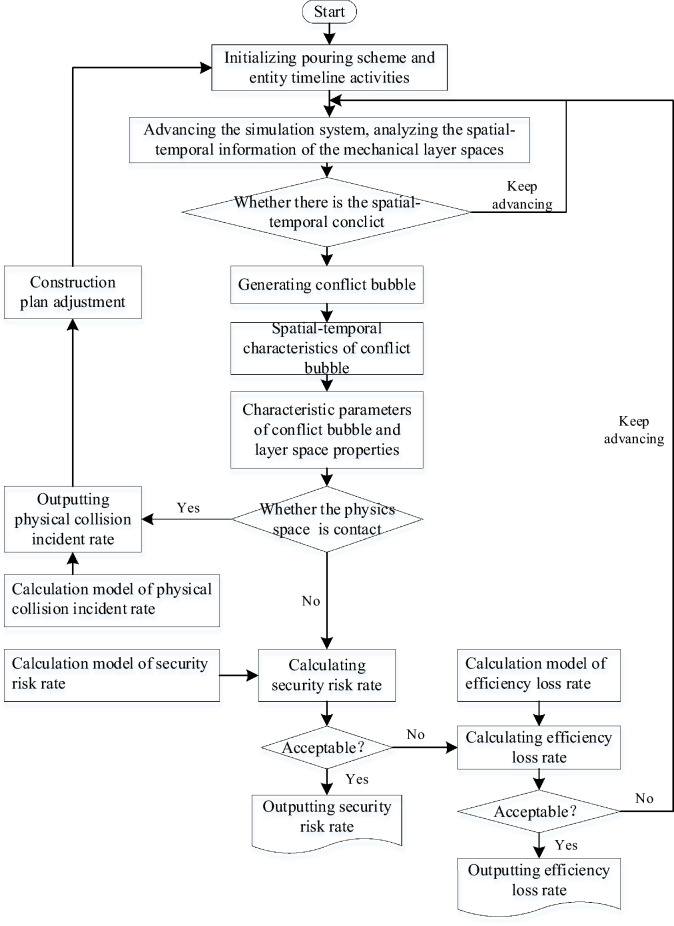
Evolution model of spatial–temporal conflict of construction machinery.

The construction machinery for the pouring block works based on the pouring plan and the respective running timeline. Through the scanning and conflict detection of the spatial–temporal state of the construction machinery, when parallel construction activities need to occupy the same space at the same time, it leads to spatial–temporal conflicts between entities, resulting in conflict bubble. When the construction machineries move according to their established trajectory and timeline, the geometric characteristics and spatial layering properties of the conflict bubble also change accordingly. The conflict bubble of construction machinery has an influence on the safety and efficiency of the construction process. The quantification of the physical collision accident rate, security risk rate, and efficiency loss rate during the evolution of spatial–temporal conflict is used as a reference. Figure 10 shows the evolution model of the spatial–temporal conflict of construction machinery.

#### Simulation model of the pouring construction system considering the spatial–temporal conflict of construction machinery

The simulation of the pouring construction system focuses on the quantification of the mechanical spatial–temporal conflict risk rate during the pouring construction process and the system simulation analysis under various operating conditions, as well as the factors affecting the spatial–temporal conflict risk rate and quantifying the spatial–temporal conflict risk rate for the whole pouring process. In the process of system simulation, the pouring of concrete is the dominant entity, which is the active event of the system. The simulation clock is selected to advance with the concrete pouring event through the scanning of the operation process of construction machinery. Then, the spatial–temporal conflict risk rate between construction machineries can be quantified. The system adopts the dominant entity clock scanning method to advance the simulation clock.

Each section strip of the pouring block is scanned, the current activity is determined according to the unloading state of the cable machine, and then the corresponding activity subroutine is carried out to continuously advance. The minimum sub-clock is further selected, the CSM and the CVM begin construction work, and the simulation of the cyclic construction operation is repeated until the end. In the above basic process, when the cyclic construction operation simulation of the construction machinery with the minimum sub-clock is promoted, it is necessary to transfer the spatial–temporal conflict quantification module of the construction machinery. The risk rate of the spatial–temporal conflict of the construction machinery in each stage and the state during the pouring construction process can be obtained.

**Fig. 11:**
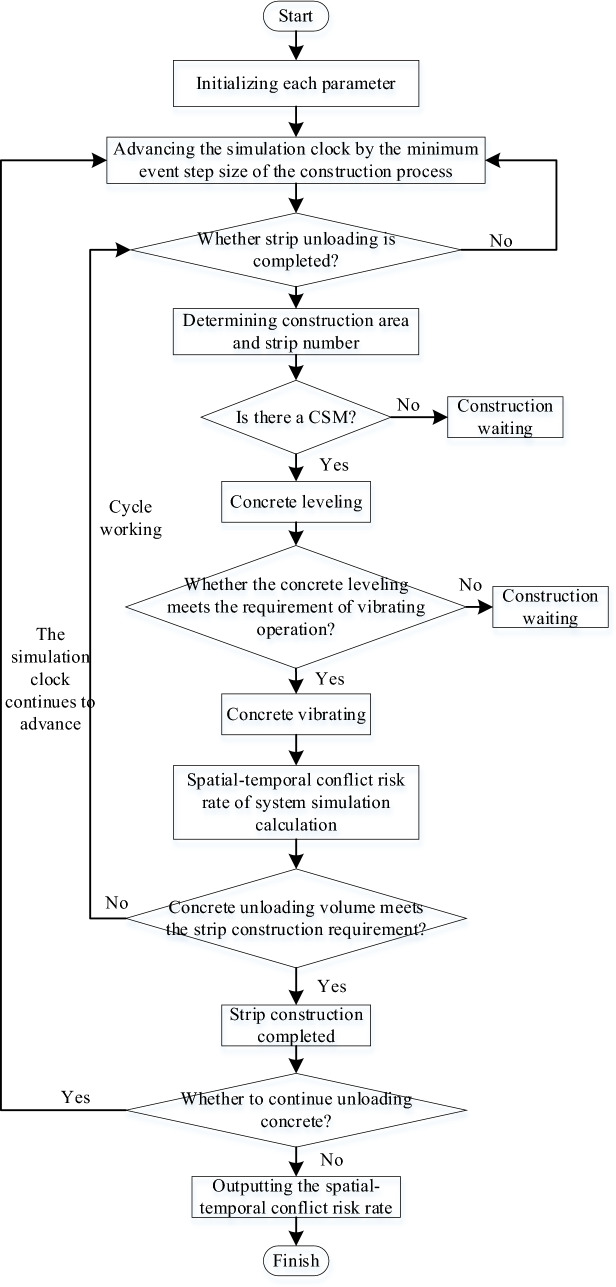
Simulation model of the pouring construction system considering the spatial–temporal conflict of construction machinery.

**Fig. 12:**
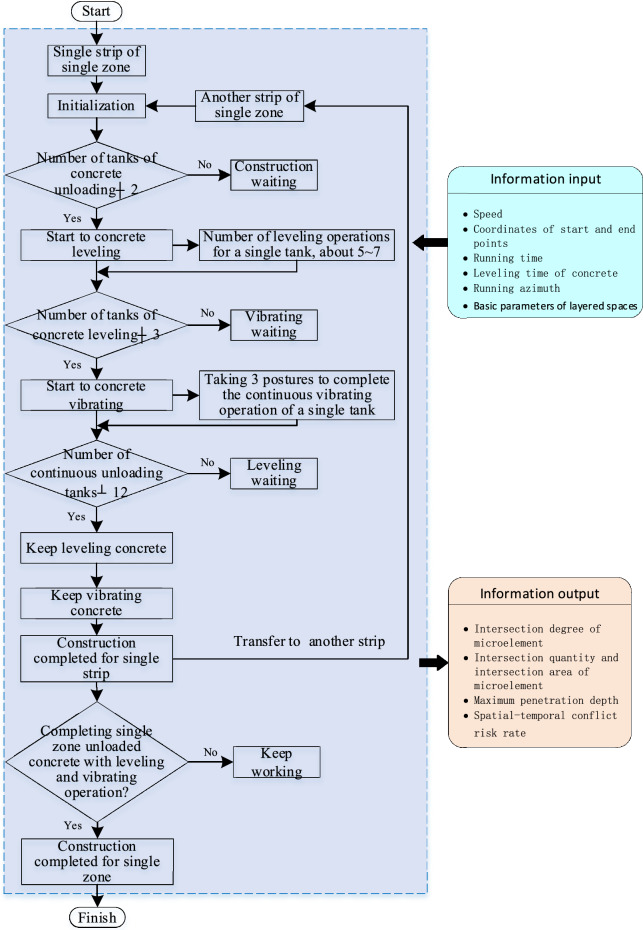
Strip construction simulation model of pouring construction system.

Based on this, the simulation model of the pouring construction system considering the spatial–temporal conflict of the construction machinery is constructed, as shown in Fig. 11. To advance the system simulation, a series of system active events that can be triggered without external conditions need to be established. The unloading of the cable machine is an active event. The unloading time of the cable machine obeys a uniform distribution. The CSM and the CVM are trigger events, and the triggering conditions are the completion of the concrete unloading and the completion of the concrete leveling. Figure 12 shows the simulation model of the strip pouring construction system.

Calculation model for the construction time of pouring block, operation characteristic model of construction machinery, the model of the correlation relationship between the unloading point and the material position of the CSM, simulation working conditions, and working conditions for calculating the safety risk rate and efficiency loss rate threshold are shown in Table 1,2,3,4,5. As shown in Table 1 (a, b) are respectively the schematic diagrams of the construction operation for the CSM and CVM. As shown in Table 3 (a, b) are respectively the schematic diagram of the relationship model between the unloading point and the waiting position and the starting area range of the pushing material for the CSM.

**Table 1:** Calculation model for the construction time of pouring block.

Cable crane unloading service time model	${T}_{unload}={e}^{u+\sigma *\sqrt{-2\text{ln}{u}_{2}}\text{*cos}(2\pi {u}_{1})}$; ${T}_{unload}$ represents the unloading service time of the cable crane; ${u}_{1}$ and ${u}_{2}$ is a random number uniformly distributed on the interval [0, 1]; $u$ is the mean of the standard normal distribution, $\sigma$ is the standard deviation of the standard normal distribution, which can take values of 7 and 0.45 respectively	
Service time model for concrete spreading operations	${T}_{s}=\left(\frac{{l}_{ul}+{l}_{l}}{{v}_{1}}+\frac{{l}_{ul}+{l}_{l}}{{v}_{2}}+{t}_{c}\right)*C$; ${T}_{s}$ represents the service time for concrete spreading operations; ${v}_{1}$ represents the pushing speed of the forward movement of the CSM; ${v}_{2}$ represents the retracting and leveling speed of the CSM; $C$ represents the number of forward and backward operation cycles (operation intensity) of the single-tank material CSM; ${t}_{s}$ represents the possible time spent on operations such as gear shifting or direction adjustment of the CSM. Referring to the operation process of the silo leveling machine at the construction site, the values of ${v}_{1}$ and ${v}_{2}$ can be between 0.5 m/s and 1.5 m/s; $C$ can take values ranging from 5 to 7; The value of ${t}_{c}$ is 1 to 3 s Constraint condition $Q={\int }_{0}^{0.5}{h}_{a}{\int }_{2}^{4.5}{w}_{l}{\int }_{o}^{7}\frac{L}{2}$; $Q$ represents the amount of concrete unloaded from the suspended tank; ${h}_{a}$ represents the thickness of the concrete spreading; ${w}_{l}$ represents the spreading width of a tank of material; $L$ is the paving length, and its expression is $L={l}_{ul}+{l}_{l}$。	(a) The schematic diagram of construction operation for the CSM
Service time model for concrete vibrating operations	${T}_{v}={t}_{m}+{t}_{a}+{t}_{v}+{t}_{r}$(Single vibration); ${T}_{v}$ represents the vibration operation time, which is measured based on the compaction time of a single concrete vibration. ${t}_{m}$ represents the time of moving to position, ${t}_{a}$ represents the time for adjusting the mechanical attitude, ${t}_{v}$ represents the compaction time of vibration, and ${t}_{r}$ represents the time for recovering the boom; ${S}_{v}=\frac{Q}{{h}_{a}}$ (The range of vibrating compaction operation after paving); ${S}_{v}$ is the sum of the length and width of the vibrating compaction area, with the length and width denoted as ${l}_{v}$ and ${w}_{v}$ respectively. $Q$ is the unloading strength of the single lifting tank, and ${h}_{a}$ is the layer thickness of the completed cast billet. The number of vibration cycles required to complete the concrete vibrating compaction task of a single hoist tank can be determined as ${N}_{v}$, expressed as ${N}_{v}=\lceil\frac{{S}_{v}}{{L}_{v}*{W}_{v}}\rceil$	(b) The schematic diagram of construction operation for the CVM

**Table 2:** Operation characteristic model of construction machinery.

Operation characteristic model of the CSM	$$v=\left\{\begin{array}{c}{a}_{1}t\\ {v}_{1}\\ {v}_{1}-{a}_{1}t\end{array}\right.\text{among }0<t\le {v}_{1}/{a}_{1}$$; $v$ represents the three-stage operation characteristic mode of uniform acceleration, uniform speed and uniform deceleration during the unidirectional operation of the CSM, ${a}_{1}$ is a constant, ${v}_{1}$ represents the uniform movement speed of the CSM
Operating characteristic model of the CVM	The operating characteristics of the CVM are set to conform to uniform distribution, and the speed range is [0.4 m/s, 0.6 m/s]

**Table 3:** The model of the correlation relationship between the unloading point and the material position of the CSM.

The correlation model between the unloading point and the waiting position of the CSM	$(\sqrt{{{(x}_{csm}-{x}_{c})}^{2}+{{(y}_{csm}-{y}_{c})}^{2}}-\sqrt{\frac{3Q}{\pi {h}_{c}}})\in \left[{R}_{1},{R}_{2}\right]$; Set the coordinates of the unloading point of the cable crane as $({x}_{c},{y}_{c})$, the amount of concrete in each tank as $Q$, the shape after unloading as a regular cone, and the height as ${h}_{c}$; Set the coordinates of the waiting position of the silo leveling machine as $({x}_{csm},{y}_{csm})$, and the safe distance interval between the waiting position of the silo leveling machine and the outer circle of the material pile is $\left[{R}_{1},{R}_{2}\right]$, where ${R}_{1}$ and ${R}_{2}$ are positive real numbers	(a) The schematic diagram of the relationship model between the unloading point and the waiting position of the CSM
Mathematical model of the starting coordinate of the CSM	; Set the coordinates of the unloading point of the cable crane as $({x}_{c},{y}_{c})$, the amount of concrete in each tank as $Q$, the shape after unloading as a regular cone, and the height as ${h}_{c}$; Set the coordinates of the waiting position of the silo leveling machine as $({x}_{csm},{y}_{csm})$, and the safe distance interval between the waiting position of the silo leveling machine and the outer circle of the material pile is $\left[{R}_{1},{R}_{2}\right]$, where ${R}_{1}$ and ${R}_{2}$ are positive real numbers	(b) The schematic diagram of the starting area range of the pushing material for the CSM

**Table 4:** Simulation working conditions.

Cable crane unloading method	Two unloading conditions of the cable crane are set. One is fixed two-point unloading, and the other is dynamic multi-point unloading according to the progress of the concrete spreading operation
The operating speed of the CSM	Under the condition that the running trajectory and the state of the construction machinery are the same, three different running speeds of the CSM are set, namely low speed 0.5 m/s, medium speed 1 m/s, and high speed 1.5 m/s
The running trajectory of the CSM	For a specific concrete tank, on the premise of maintaining the running speed and construction state unchanged, four running trajectories of the CSM were considered by randomly generating the starting and ending points of the CSM
The operating mode of the CVM	The movement and construction process of the CVM is simplified to a low uniform speed operation state, and the operation speed is set at 0.5 m/s

**Table 5:** Working conditions for calculating the safety risk rate and efficiency loss rate threshold.

Taking the reference point of the projection plane of the CSM as the origin of the rectangular coordinate system, the projection plane moves along the positive Y-axis. The projection plane of the CVM moves respectively at angles of 0°, 45°, 90°, 135°, and 180° with the reference point. It is stipulated that the clockwise direction of the projection plane of the CSM is positive. The contact states between two types of machinery under different working conditions are defined as point contact, edge contact (or point-edge contact)		
Safety risk rate	${S}_{T}=\frac{1}{10}({S}_{p}^{0}+{S}_{l}^{0}+{S}_{p}^{45}+{S}_{l}^{45}+{S}_{p}^{90}+{S}_{l}^{90}+{S}_{p}^{135}+{S}_{l}^{135}+{S}_{p}^{180}+{S}_{l}^{180})$	${S}_{T}$ and ${E}_{T}$ are the thresholds of the security risk rate and the efficiency loss rate respectively; ${S}_{p}^{0}$, ${S}_{l}^{0}$, ${S}_{p}^{45}$, ${S}_{l}^{45}$, ${S}_{p}^{90}$, ${S}_{l}^{90}$, ${S}_{p}^{135}$, ${S}_{l}^{135}$, ${S}_{p}^{180}$, ${S}_{l}^{180}$ and ${E}_{p}^{0}$, ${E}_{l}^{0}$, ${E}_{p}^{45}$, ${E}_{l}^{45}$, ${E}_{p}^{90}$, ${E}_{l}^{90}$, ${E}_{p}^{135}$, ${E}_{l}^{135}$, ${E}_{p}^{180}$ and ${E}_{l}^{180}$ respectively represent the safety risk rate and efficiency loss rate thresholds in the point-point contact and edge contact (or point-edge contact) states under the five working conditions of 0°, 45°, 90°, 135°, and 180°
Efficiency loss rate threshold	${E}_{T}=\frac{1}{10}({E}_{p}^{0}+{E}_{l}^{0}+{E}_{p}^{45}+{E}_{l}^{45}+{E}_{p}^{90}+{E}_{l}^{90}+{E}_{p}^{135}+{E}_{l}^{135}+{E}_{p}^{180}+{E}_{l}^{180})$	

## Case study

Taking a typical pouring block of the Baihetan arch dam as an example, according to the research content such as the quantification method of the spatial–temporal conflict of construction machinery and the simulation model of the pouring operation system considering the spatial–temporal conflict of the construction machinery, the spatial–temporal conflict simulation and visual analysis system of the construction process for the pouring block is constructed. This can achieve visual simulation analysis for the construction process of a single pouring block. Taking the construction activity in the pouring block as the smallest unit, applying computer simulation technology to carry out pre-simulation and dynamic visualization rehearsal of the pouring construction process, the potential spatial–temporal conflict between the construction machineries can be found, which can improve construction safety and efficiency. Additionally, by designing a scientific and reasonable spatial–temporal conflict adjustment mechanism, the dynamic deployment of the construction machinery and the refined and scientific construction organization and management can be realized based on adopting effective plan adjustment and optimization strategies.

### Background information

**Fig. 13:**
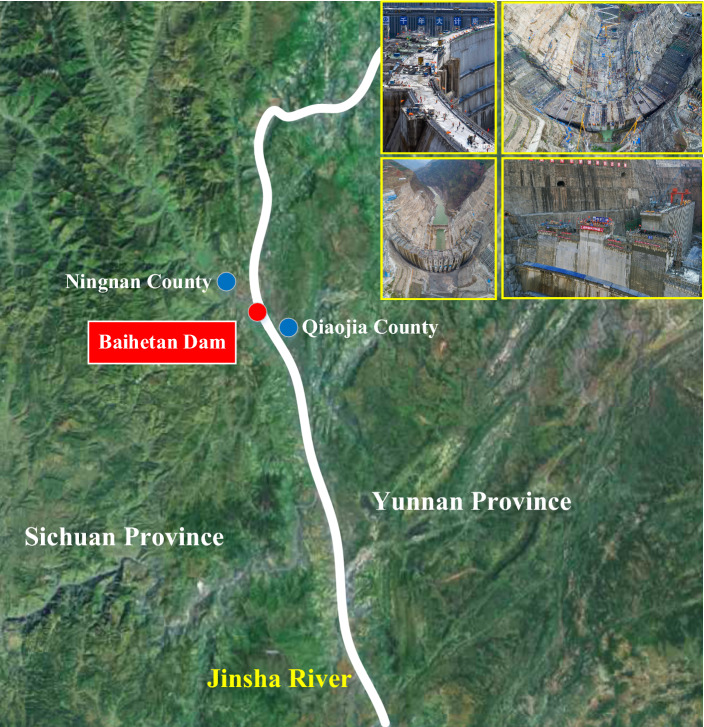
Geographical location map of Baihetan dam. (Source from: https://map.baidu.com/search/%E7%99%BD%E9%B9%A4%E6%BB%A9%E6%B0%B4%E7%94%B5%E7%AB%99/@11468650.790579714,3157682.2773694755,10.28z/maptype%3DB_EARTH_MAP?querytype=s&da_src=shareurl&wd=%E7%99%BD%E9%B9%A4%E6%BB%A9%E6%B0%B4%E7%94%B5%E7%AB%99&c=32&src=0&pn=0&sug=0&l=10&b=(11328293.866916457,3014104.371983306;11591172.4123784,3146696.7350189337)&from=webmap&biz_forward=%7B%22scaler%22:2,%22styles%22:%22sl%22%7D&device_ratio=2, @ 2025 Baidu – GS(2023)3206; Microsoft® Visio® 2021MSO (version 2502 Build 16.0.18526.20144) 64-bit).

The Baihetan Hydropower Station on the Jinsha River is located in the lower reaches of the Jinsha River bordering Ningnan County, Sichuan Province and Qiaojia County, Yunnan Province. The capacity reaches 16 million kW, and the average annual power generation is 62.443 billion kWh. This project is the third tallest arch dam project in China and consists of main buildings such as a barrage, a flood discharge and energy dissipation structure, and a water diversion and power generation system. The barrage is a concrete double-curvature arch dam with a crest elevation of 834 m and a maximum dam height of 289 m. The arch dam is divided into 31 dam sections, with a water cushion pond and an Erdao dam under the dam^2^. As shown in Fig. 13 is the Geographical location map of Baihetan dam.

In this research, relying on the Baihetan arch dam distributed optical fiber temperature monitoring and forecast feedback project, the typical and real construction process of the pouring block in the construction site can be mastered. Based on this, the spatial–temporal quantification method and system simulation model of the construction machinery investigated in this research are applied to the pouring construction process of a typical pouring block for the Baihetan arch dam. This is expected to achieve the scientific, efficient, and refined construction machinery dynamic allocation and the construction organization and management of the pouring block, which can effectively avoid the spatial–temporal conflict of the construction machinery and improve the safety and efficiency of the construction process. Furthermore, the investigated content described above can be applied to other arch dam construction projects.

**Fig. 14:**
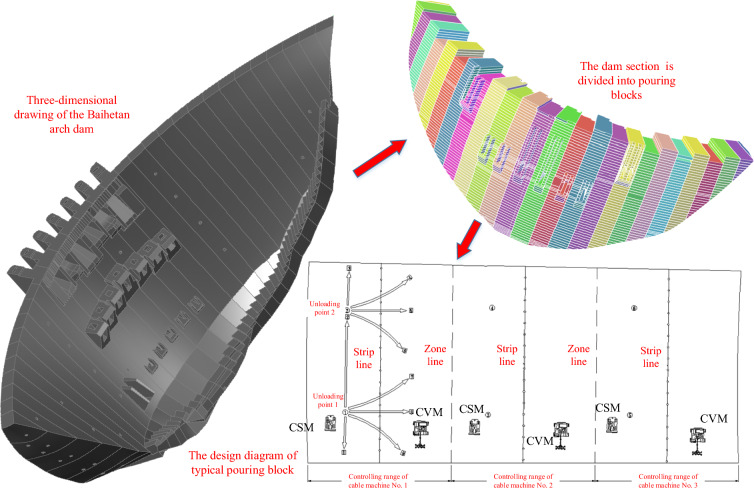
Three-dimensional drawing of the Baihetan arch dam and the design diagram of a typical pouring block.

Figure 14 shows a three-dimensional drawing of the Baihetan arch dam and the design diagram of a typical pouring block. The typical pouring block is a flat block with a size of 54 m × 24 m (length × width), and the thickness of the pouring block is 3 m. The pouring block is divided into six pouring layers for continuous construction. Adopting the strip construction method, the concrete pouring construction is divided into three zones and six strips. Each zone is 18 m long, and each zone is equipped with a cable machine, a CSM, and a CVM.

### Simulation boundary conditions and related construction parameters

The simulation boundary conditions and related construction parameters mainly include the following five aspects.

Information about the typical pouring block: Taking the pouring surface as the base plane, the river direction, the transverse river direction, and the pouring thickness direction are defined as the X direction, the Y direction, and the Z direction, respectively. The typical pouring block is divided into three zones and six strips, which are numbered as zone 1, zone 2, and zone 3. The corresponding areas of each strip in each zone are numbered 1–1, 1–2, 2–1, 2–2, 3–1, and 3–2.

Information about the decomposition of the construction process: During the pouring construction process, the whole construction process is broken down according to the number of times concrete is unloaded. The construction operation method of concrete pouring adopts a sub-partition and a sub-strip, and the concrete pouring quantity of each strip can be determined by calculation. A 9 m^3^ capacity hanging tank is used for concrete transportation in the Baihetan project. The number of unloading tanks can be used as the basis for the decomposition of the construction process. Notice that the CSM needs two regular cans of concrete to begin its operation, and these two cans are considered as one large can for easy calculation. The calculation formula can be shown as Eq. (3):

3 $$p\left(k\right)=\left\lceil\left(\frac{{l}_{a}*{w}_{a}*{h}_{a}}{Q}-1\right)\right\rceil$$

where $p\left(k\right)$ is the number of times concrete is unloaded, ${l}_{a}$ is the length of a single strip along the river, ${w}_{a}$ is the width of a single strip across the river, ${h}_{a}$ is the pouring thickness of a single layer, and $Q$ is the amount of concrete loaded in each tank.

Information about the layered space of construction machinery: The physical space of the CSM is always fixed, while the physical space of the CVM changes with the working state. In the process of system simulation, the layered space state of the CSM and CVM is determined by their running speeds and posture. Specifically, for the CVM, seven working statuses are set, covering the working statuses of short-range vibrating, medium-range vibrating, and long-range vibrating. Figure 14 shows the infographic of a typical pouring block, the decomposition of the construction process, and the layered space of the construction machinery.

**Fig. 15:**
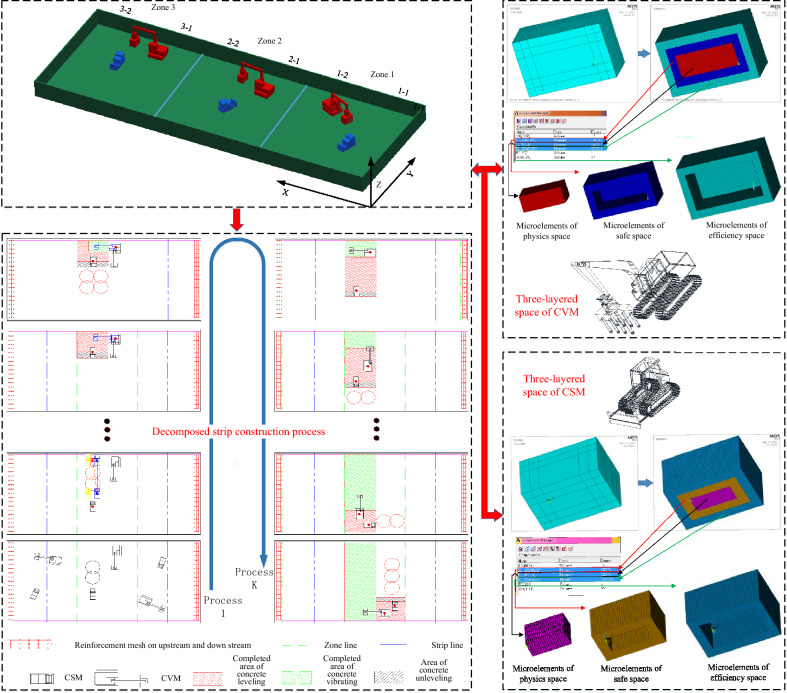
Infographic of a typical pouring block, decomposition of the construction process, and layered space of construction machinery.

**Fig. 16:**
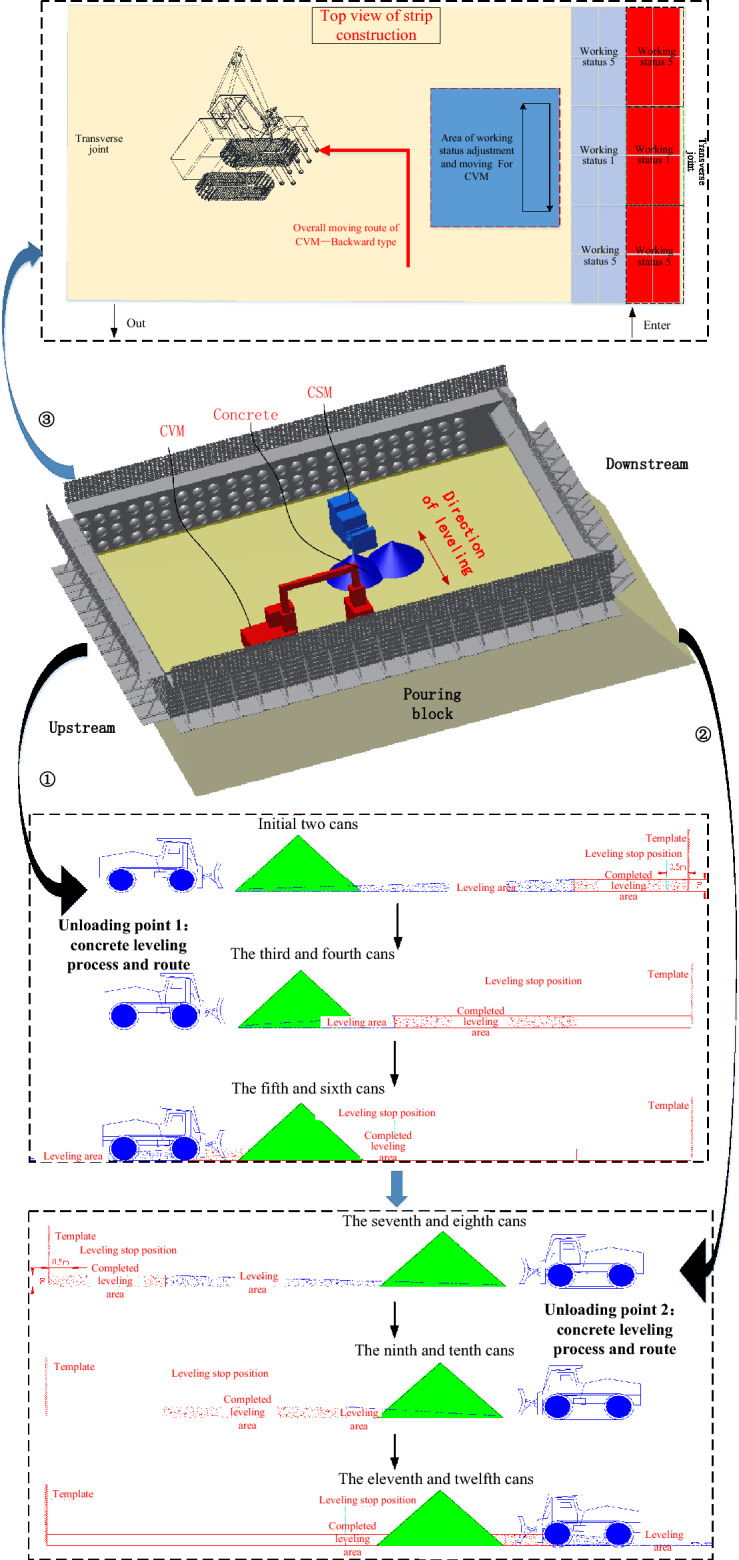
Schematic diagram of the construction process of leveling and vibrating on a single strip.

This section provides information about the working intensity and operation process for the CSM and the CVM. Figure 16 shows the schematic diagram of the construction process of leveling and vibrating on a single strip. The leveling operation of the CSM is simplified into a linear leveling process, and the leveling position priority is sequentially leveled from one side of the horizontal seam to the other side of the horizontal seam. According to the parameters of the bucket pushing capacity, the amount of concrete loaded in a single hanging tank, the distance between the starting point and the end point, and other parameters, it is determined that the number of times that the CSM completes the leveling of the single hanging tank concrete is 5–7 times.

The triggering condition for the vibrating operation is that the CSM completes the leveling task of a certain volume or a certain area of concrete, and the entry of CVM is not disturbed by other disturbances. According to the actual pouring situation at the construction site of the Baihetan arch dam, after the CSM completes the leveling task of the concrete for the two initial hanging tanks, the CVM can enter the site and prioritize the completion of the vibrating of the concrete at the template position near the transverse joint surface.

Simulation boundary conditions and related construction parameters, and system definition are shown in Tables 6 and 7.

**Table 6:** Simulation boundary conditions and related construction parameters.

Parameters related to the stratified space of construction machinery	Considering the characteristics of the pouring construction activities of the block surface and the difficulty of the system simulation calculation, combined with the actual situation of the construction site of the Baihetan project, the calculation model is reasonably simplified. Some variables in the mathematical model are taken as fixed values. Among them, taken as 0 m/s to 1.5m/s, is taken as 0.5s to 1.5s, and the constant is taken as 0.5m. is taken as 0 m/s to 1 m/s, is taken as 0.5s to 1.5s, the constant is taken as 0.5m, the safety factor is set within the range of 1.5 to 2, the reaction time is set within the range of 0.4 to 0.6s, and the operating speed during tank loading into the pouring block and alignment is taken as 1m/s. It is assumed that the safe space of the CSM and the CVM is only affected by their operating speed.											
	(a) Physical spatial parameters table of CSM (Unit: m)											
			Length (longitudinal)				Width (horizontal)				Height (vertical)	
	CSM		4.3				1.9				2.8	
	(b) Physical spatial parameters table of CVM in multiple working states (Unit: m)											
			Number		Length (longitudinal)		Width (horizontal)				Height (vertical)	
	CVM		Status 0		5.8		3				4.4	
			Status 1		6.2		3				4.2	
			Status 2		7.6		3				3.7	
			Status 3		8.8		3				3.2	
			Status 4		5.4		4				4.2	
			Status 5		6.2		4.5				3.7	
			Status 6		7		5				3.2	
	(a) and (b) show the physical spatial parameters of the CSM and the CVM in multiple working states, respectively. Among them, states 1 to 3 correspond to the working states of short-distance vibration without deflection of the rotating arm, medium-distance vibration, and long-distance vibration, respectively. States 4 to 6, respectively, correspond to the short-range, medium-range, and long-range vibration working states when the rotating arm is deflected by approximately 45°											
The discharge volume of the cable crane and the coordinate parameters of the discharge of each zone and each belt	To simplify the unloading process of the cable crane, the concrete materials produced by the production system and transported by the cable crane are assumed to be concrete with a single grade distribution. The concrete production and transportation planning before the construction is not considered. The unloading volume of the cable crane is measured according to the number of hoisting tanks transported. (c) shows the coordinate parameters of the discharge points of each zone and each belt under the two-point discharge condition and the discharge volume of each discharge point. In addition, the multi-point unloading conditions that change dynamically along with the operation process of the CSM are set. (d) shows the planar coordinates of the unloading points and the unloading volume of each zone and each belt under the multi-point unloading conditions											
	(c) Planar coordinates of unloading points and unloading quantities of each belt in each zone (Unloading conditions at two fixed points)											
	Zone		Belt		Coordinate of unloading point 1		Unloading point 1 Unloading volume, unit: tank		Coordinate of unloading point 2		Unloading point 2 Unloading volume, unit: tank	
	Zone 1		1–1		(4.5, 8)		6		(4.5, 16)		6	
			1–2		(13.5, 8)		6		(13.5, 16)		6	
	Zone 2		2–1		(22.5, 8)		6		(22.5, 16)		6	
			2–2		(31.5, 8)		6		(31.5, 16)		6	
	Zone 3		3–1		(40.5, 8)		6		(40.5, 16)		6	
			3–2		(49.5, 8)		6		(49.5, 16)		6	
	(d) Planar coordinates of unloading points and unloading quantities of each belt in each zone (Unloading conditions at multi-point)											
	Zone	Belt	Coordinate of unloading point 1	Unloading point 1 Unloading volume, unit: tank	Coordinate of unloading point 2	Unloading point 2 Unloading volume, unit: tank	Coordinate of unloading point 3	Unloading point 3 Unloading volume, unit: tank	Coordinate of unloading point 4	Unloading point 4 Unloading volume, unit: tank	Coordinate of unloading point 5	Unloading point 5 Unloading volume, unit: tank
	1	1–1	(4.5, 8)	3	(4.5, 10)	2	(4.5, 12)	2	(4.5, 14)	2	(4.5, 16)	3
		1–2	(13.5, 8)	3	(13.5, 10)	2	(13.5, 12)	2	(13.5, 14)	2	(13.5, 16)	3
	2	2–1	(22.5, 8)	3	(22.5, 10)	2	(22.5, 12)	2	(22.5, 14)	2	(22.5, 16)	3
		2–2	(31.5, 8)	3	(31.5, 10)	2	(31.5, 12)	2	(31.5, 14)	2	(31.5, 16)	3
	3	3–1	(40.5, 8)	3	(40.5, 10)	2	(40.5, 12)	2	(40.5, 14)	2	(40.5, 16)	3
		3–2	(49.5, 8)	3	(49.5, 10)	2	(49.5, 12)	2	(49.5, 14)	2	(49.5, 16)	3
Information on the serial numbers of each cable machine, the conveying cycle, and the unloading sequence	At the construction site of the Baihetan project, seven 30 T translational cable machines were arranged, adopting a double-layer layout scheme of high and low lines. When two cable machines on the same platform are close to each other, the minimum distance between the main cables is 10 m for the high platform and 12.5 m for the low platform, respectively. When two cabling machines on different platforms are in operation, the minimum safe distance between the main cables is 6 m. Based on the actual size of the pouring block and the relevant information of the division and strip, combined with the reference value of the safe distance between two cable machines or hoisting tanks under the operating conditions of the cable machine, it is assumed that one cable machine is matched in each of the three zones. Cable machines of different numbers have the working conditions of simultaneous material transportation and unloading. The numbers of the cable machines in the same zone are matched in sequence according to the zone numbers, that is, from the upstream side along the river to the downstream side. The first section of the pouring block is matched with the No. 1 cable machine, the second section with the No. 2 cable machine, and the third section with the No. 3 cable machine. Moreover, the transportation cycle of concrete into the block by each zone and each belt cable machine follows a normal distribution, and each numbered cable machine is synchronously unloaded in each zone and each belt											
Information on the operation intensity and running process of the CSM	The operation of the CSM begins after the cable machine unloading is completed. This paper takes the unloading of the concrete materials from the two hoisting tanks as the initial moment of the strip pouring. At this time, the CSM starts the construction operation. After that, the CSM carries out the concrete spreading operation after the concrete materials from each hoisting tank are unloaded. To meet the requirements of protecting the vibrated concrete surface, a certain thickness of concrete needs to be spread along the operation trajectory of the CSM. Based on this, the spreading operation of the CSM can be simplified as a linear spreading process. Considering the limited factors of the working surface of construction machinery in actual construction, in the simulation calculation, 0.5 m away from the transverse joint surface is taken as the termination position of pushing materials along the transverse joint surface The figure shows in detail the state changes of the initial two concrete tanks being spread by the CSM, which are decomposed into single concrete tanks and spread according to the operation intensity of the CSM. The paving direction of concrete materials for tanks 1 to 3 is from discharge point 1 to the near side transverse joint surface, and the paving direction of concrete materials for tanks 4 to 6 is from discharge point 1 to discharge point 2. When the unloading point is transferred to unloading point 2, the paving direction of the concrete for tanks 7 to 9 is from unloading point 2 to the near side transverse joint surface (consistent with the paving direction of tanks 4 to 6), and the paving direction of the concrete for tanks 10 to 12 is from unloading point 2 to unloading point 1											
Information on the working intensity and operation process of the CVM	; is the effective area of a single vibration of the vibrating rod group.. Referring to the previous values of and of 1.5 m and 1 m, respectively, is 1.5m^2^ (the vertical vibration thickness is 0.5 m, by default the vertical vibration is completed in one time and is sufficient). represents the service time of a single vibration operation. According to the previous text, the real data of the construction site was statistically analyzed, and the average value processed was taken as 45 s Based on the calculation of a concrete discharge volume of 9 m^3^ for a single suspended tank and a pouring layer thickness of 0.5 m, it takes 12 vibrations to complete the task of compacting the concrete in one suspended tank, which takes approximately 9 min											
Safety distance parameters of formwork for pouring blocks	The outermost part of the block surface pouring construction is surrounded by formwork. When the construction machinery is close to the formwork position, to avoid physical collisions and safety risks, the machinery usually needs to reserve a certain safety distance. In this paper, this distance is defined as the formwork safety distance. The concrete vibration within the formwork safety distance is carried out by manual vibration. According to the actual situation of the construction site, this safety distance is set at 0.5 m

**Table 7:** System definition.

Space	To describe the spatial position of the pouring block construction machinery in the working space, a system simulation construction coordinate system is established based on the pouring block design data. The pouring block entity is constructed as a two-dimensional plane coordinate, and the spatial vertical coordinates are successively increased for the pouring of the green layer cover. All the pouring block construction machinery moves in the two-dimensional plane. Based on this, the spatial position and orientation data information of the construction machinery in each strip can be determined. To combine the simulation construction coordinate system with the actual construction process, the system simulation construction coordinate system adopts the same coordinate system as the design documents.	
Time	The construction time system for the pouring block is mainly advanced by the natural time system, based on the construction process flow, construction methods, and the regular operation time of phased construction operations. The pouring block construction simulation system focuses on the time passage process of each construction machinery within the construction behavior time period. At the same time, the risk values of spatial-temporal conflicts caused during the construction process and the adjustment strategies of the construction plan will be taken into consideration.	
Entity	The entities within the pouring block construction system are classified into active and passive entities based on their behavioral characteristics. The description and expression of the hierarchical space of the entity space are carried out by using attributes such as active or passive attributes, geometric information, motion characteristics, operation trajectories, and process flows. Further, the entities of the pouring block construction simulation system can be classified as shown in Table 7-1, which is the entity classification table:	
	(a) Entity Classification of the simulation of the pouring block construction system	
	**Classification**	**Content**
	Block surface	The structure and dimensions of the pouring block, functional structure, etc.
	Construction machinery	CSMs, CVMs, cable machines, etc.
	Coordinate dimension	Running trajectory, node coordinates, and special structural dimensions
	Cable machine unloading	The unloading point, unloading volume, and spreading range of the cable crane
	Mechanical matching	The mechanical matching combination of the strip construction method is divided

### Development of a simulation system for pouring construction considering the spatial–temporal conflict of construction machinery

**Fig. 17:**
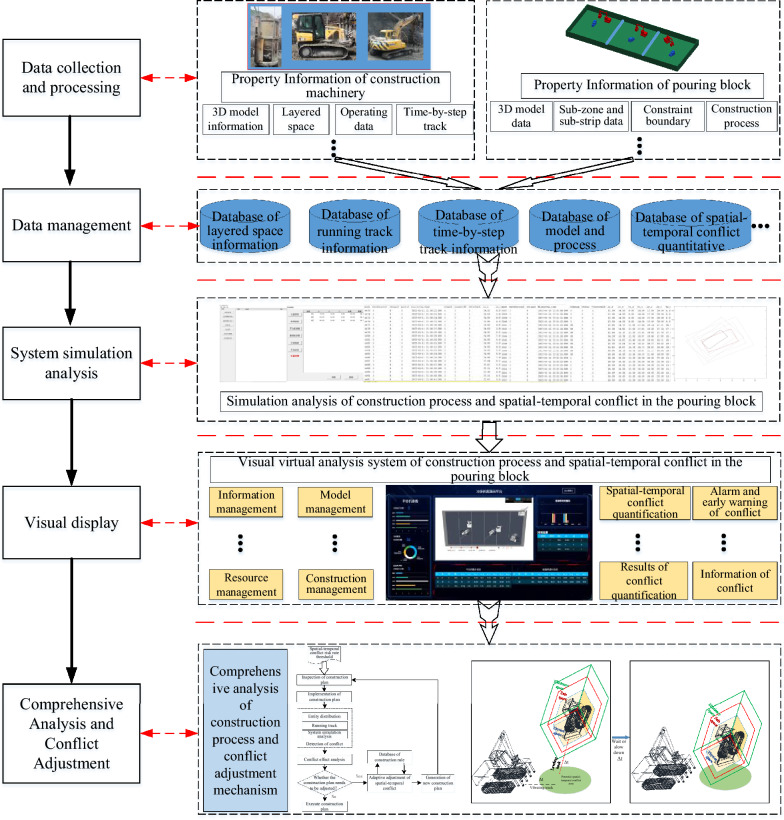
Overall frame diagram of the simulation and visualization system of pouring construction considering the spatial–temporal conflict of the construction machinery.

In this research, a simulation and visualization system is independently developed for the construction process of a pouring block considering the spatial–temporal conflict of construction machinery based on Microsoft SQL Server database technology, PowerBuilder 9.0 development tools, the Three.js 3D processing development environment, and other technology. This system mainly includes a simulation calculation module and a visual simulation module. Figure 17 illustrates the overall frame diagram of the simulation and visualization system of pouring construction considering the spatial–temporal conflict of the construction machinery.

#### Simulation calculation module of the pouring construction

For the construction mode of the sub-zone and sub-strip, the construction process of each zone is independent. The simulation process is iteratively extended to the entire pouring block based on the simulation process of strip construction. The unloading cycle of the cable machine is set as the main simulation clock, and the simulation scan time is $\Delta t$. The main program scans the current operation status, construction progress, spatial coordinates, and other factors of the construction machinery. After triggering the leveling task, the system simulates the operation of the CSM and records the running micro-step trajectory. In addition, the system simulates the operation of the CVM and records the running micro-step trajectory after triggering the vibrating task. Then, the system continues to advance until the pouring construction task of each zone is completed.

To quantify the spatial–temporal conflict between construction machineries in the system simulation process, it is necessary to construct the core datasheet structure. The database and corresponding table structure established in this research mainly include the geometric parameters of the pouring block, the parameters of the sub-zone and sub-strip, the basic parameters of the CSM and CVM, the layered space parameters, the micro-step trajectory parameters of the CSM and CVM, and the detailed parameters of the micro-steps of the CSM and CVM.

**Fig. 18:**
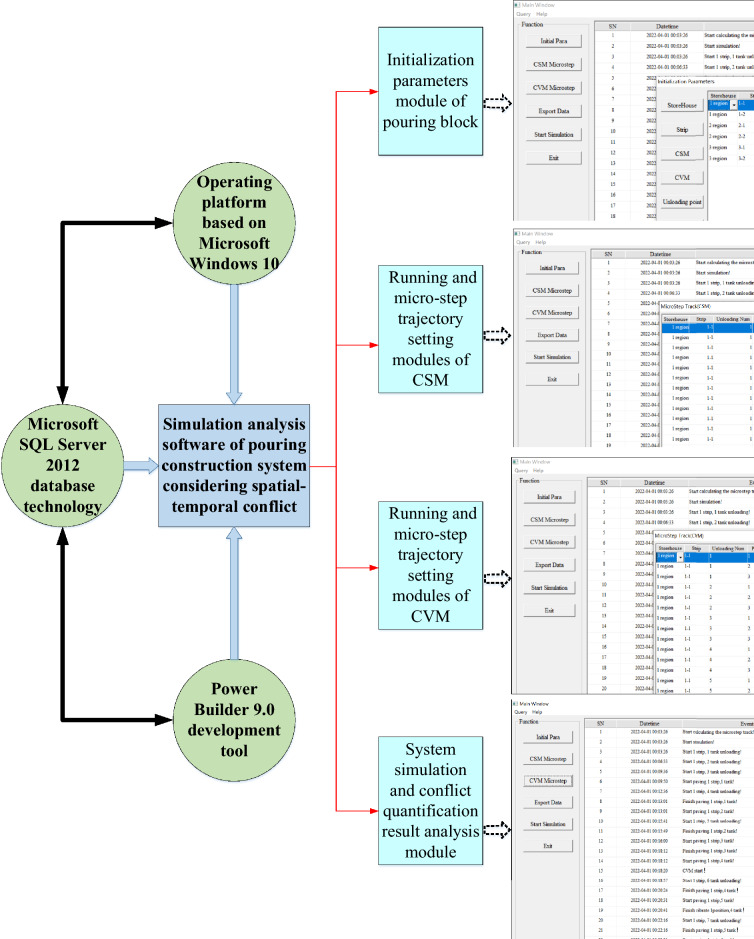
Main functions and components of the simulation calculation module of the pouring construction system.

Figure 18 shows the main functions and components of the simulation calculation module of the pouring construction system. The simulation calculation module is mainly composed of four modules: the initialization parameter module, the trajectory setting modules of the CSM and CVM, and the analysis module of the simulation result. The initialization parameter module includes the parameter settings of the pouring block, strip parameter settings, parameter settings of the CSM and CVM, and other parameter settings. The trajectory setting modules of the CSM mainly simulate the running and working processes of the CSM. According to the coordinates of the starting and ending positions, the simulation of the leveling operation is completed and the micro-step trajectory of the CSM is stored in the database. Similar to the trajectory setting modules of the CSM, the trajectory setting modules of the CVM mainly simulate the running and working processes of the CVM. According to the coordinates of the starting and ending positions, the simulation of the vibrating operation is completed and the micro-step trajectory of the CVM is stored in the database. The analysis module of the simulation result can calculate and generate the time-by-step nodes and quantify the physical collision accident rate, security risk rate, and efficiency loss rate at any time or period based on the quantification theory of spatial–temporal conflict between construction machines.

#### Visual simulation module of spatial–temporal conflict between construction machineries

**Fig. 19:**
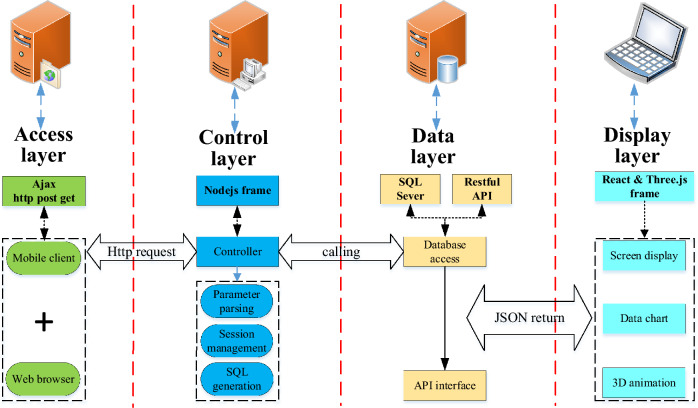
Architectural diagram of the visualization simulation module of spatial–temporal conflict between construction machineries.

The visual simulation module of spatial–temporal conflict between construction machineries adopts a B/S structure and a three-layer development system, the main functions of which include data reading and management, data visualization display, data dynamic management, visual output, and related report functions. Figure 19 shows the architectural diagram of the visualization simulation module of spatial–temporal conflict between construction machineries.

**Fig. 20:**
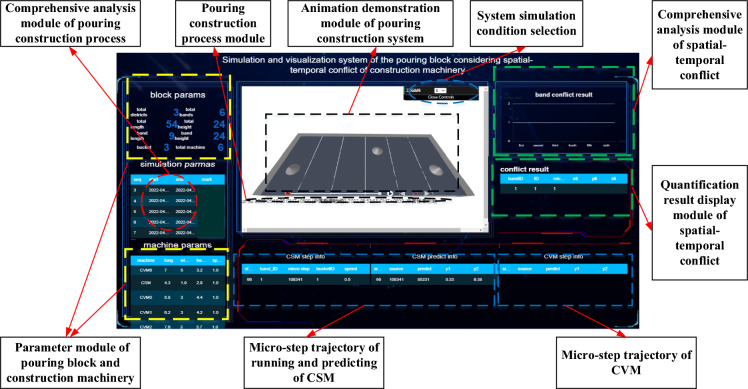
Main menu interface and function display diagram of the foreground display module of the visual simulation module of spatial–temporal conflict of construction machinery.

Figure 20 shows the main menu interface and function display diagram of the foreground display module of the visual simulation module of the spatial–temporal conflict of the construction machinery.

### Spatial–temporal conflict analysis of construction machinery in pouring block

The spatial–temporal conflict risk of the construction machinery in the pouring block may lead to collision accidents, security risks, and efficiency losses. The accumulation of spatial–temporal conflict of construction machinery in hundreds of continuous pouring blocks may cause a series of problems, such as subsequent construction interference, construction progress lag, and construction management difficulty, because the construction safety and efficiency of a dam are associated with those of each pouring block. With the quantitative spatial–temporal conflict risk rate between construction machineries at each period or time node, the running state of the construction machinery and the spatial–temporal conflict risk rate can be displayed in real time. In the case of a clear physical collision risk rate or the threshold values of the security risk and efficiency loss rate caused by spatial–temporal conflict, a comparative analysis is made to determine the risk effect, and timely corrections and spatial–temporal conflict adjustments are made.

#### Quantification results and influence effect analysis of the spatial–temporal conflict of construction machinery according to the decomposition of the number of times concrete is unloaded

Concerning the actual construction parameters on site, each strip is unloaded 12 tanks concrete, and each tank concrete is decomposed into 24 micro steps in the concrete spreading process (including the process of forward and backward). To calculate the quantitative value of the spatial–temporal conflict risk of construction machinery under each micro-step, the quantification results and influence effect analysis of the spatial–temporal conflict of construction machinery are processed according to the decomposition of the number of times concrete is unloaded.

**Fig. 21:**
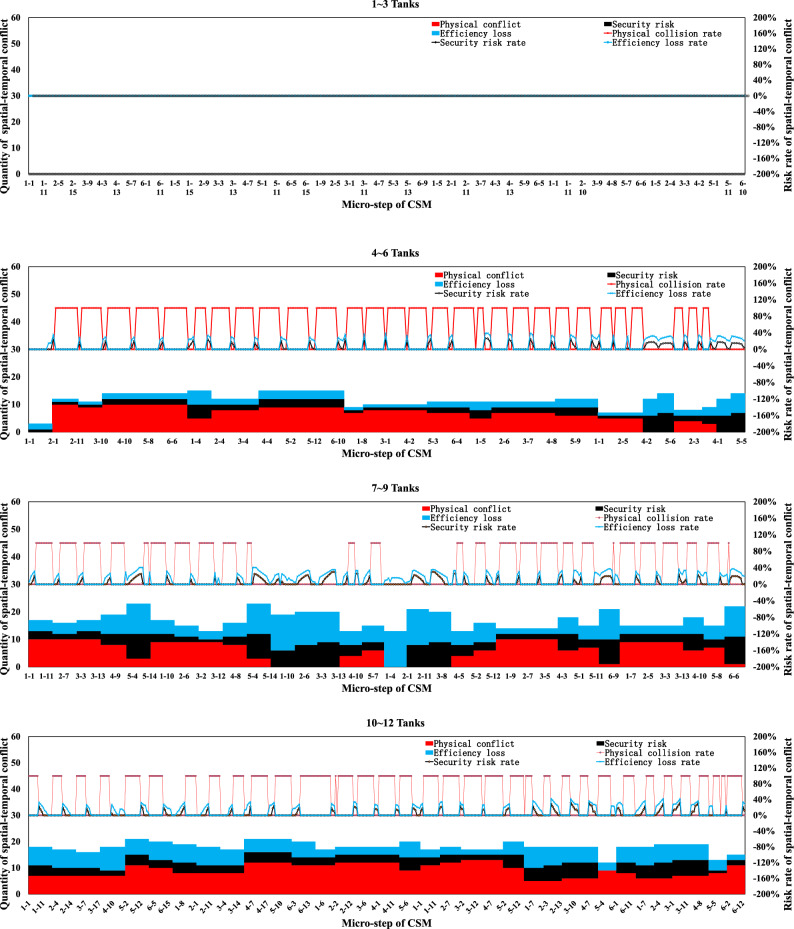
Quantification results of spatial–temporal conflict of construction machinery at each micro-step for pouring block.

**Fig. 22:**
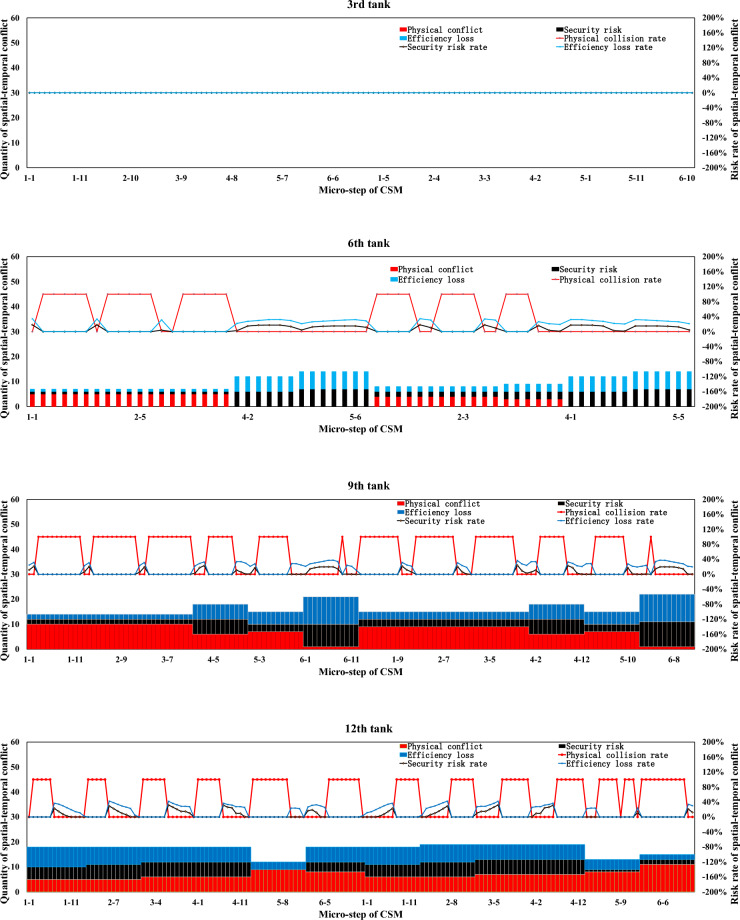
Statistical results of spatial–temporal conflict of typical unloading tank times.

The physical collision statistics, security risk statistics, and efficiency loss statistics are introduced to evaluate the amount of conflict during the construction period. Figure 21 shows the quantification results of the spatial–temporal conflict and the amount of spatial–temporal conflict of the construction machinery at each micro-step (per second) for the pouring block. Figure 22 shows the statistical results of the spatial–temporal conflict of typical number of times concrete is unloaded (3rd, 6th, 9th, and 12th). The horizontal coordinate represents the micro-step of the CSM after the unloading of concrete in each tank for each belt (for example 1–1 ~ 1–24……6–1 ~ 6–24); The principal ordinate represents the number of physical conflicts, security risks, and efficiency losses that occur; The secondary ordinates represent values for physical conflicts, safety risks, and efficiency losses. It can be seen from the figure that: (1) In the current simulation boundary conditions, the physical collision accident mainly occurs in the leveling and vibrating process when concrete is unloaded 4–11 times for the typical zone. When the CVM participates in the construction process, the occupation of all the construction space resources is aggravated in the strips, and the physical collision statistics are significantly intensified during the leveling and vibrating operations of the 9th, 10th, and 11th tanks. (2) In the current simulation boundary conditions, the security risk and efficiency loss caused by spatial–temporal conflict run through the whole strip construction process. It can be seen that the spatial–temporal conflict problem always exists in the pouring construction process, and the problems of safety risk and efficiency loss are inevitable. (3) In view of the physical collision accidents caused by the spatial–temporal conflict in the strip construction process, the running track and state of the CSM and CVM entering the strip at the same time should be considered, and the approach time and running route should be reasonably adjusted. In addition, it is necessary to consider the movement trajectory of the construction machinery and the order of movement to reasonably avoid a conflict risk.

#### Quantification results and influence effect analysis of the spatial–temporal conflict of construction machinery according to the decomposition of strips

**Fig. 23:**
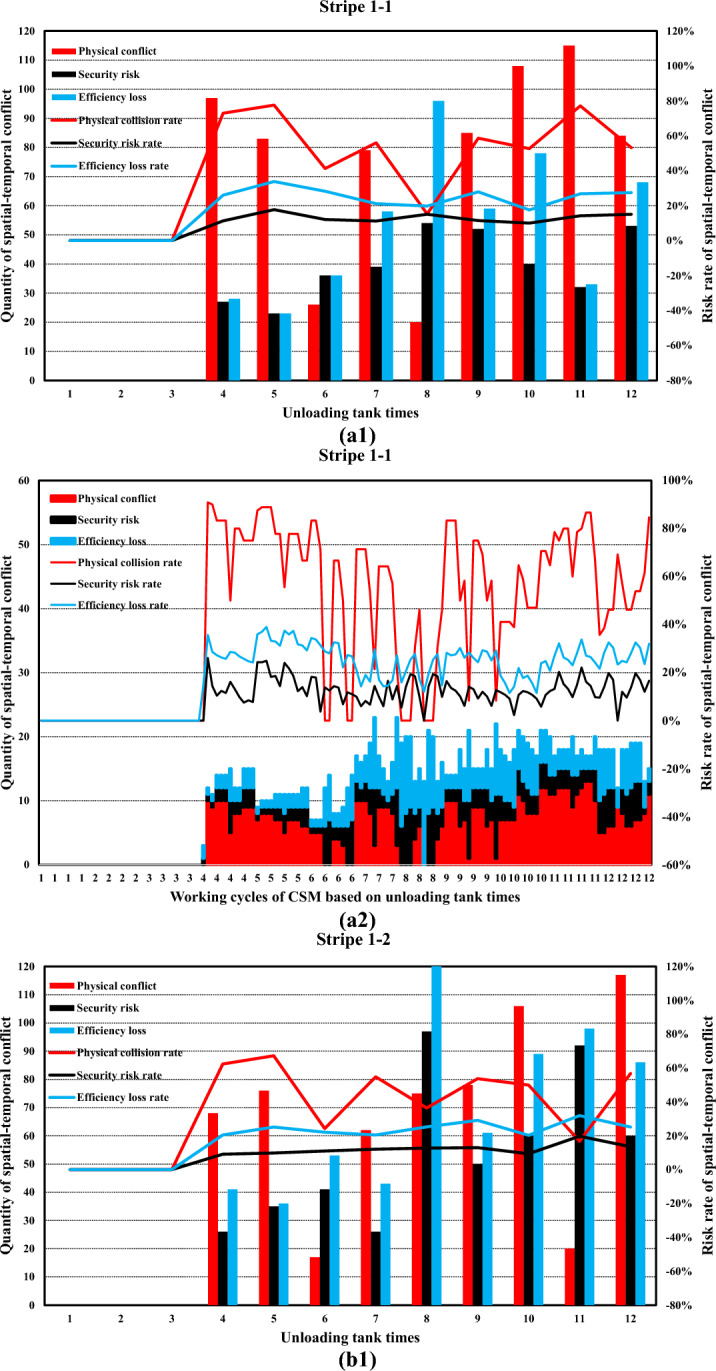

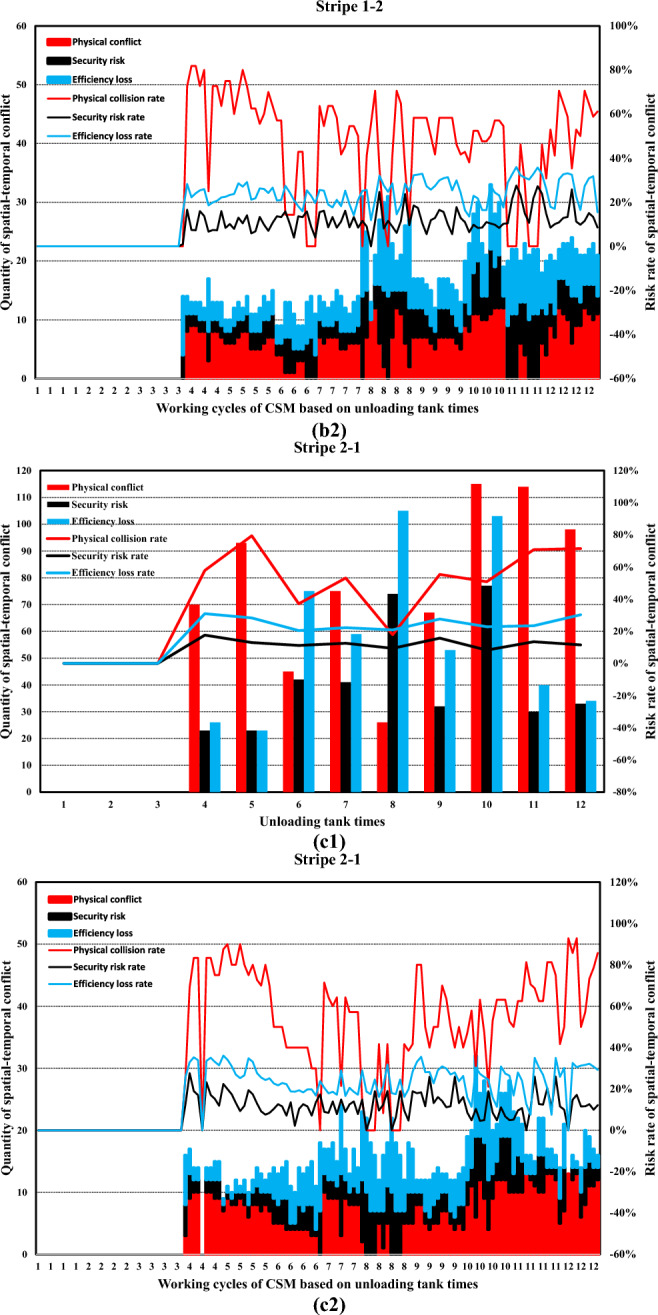

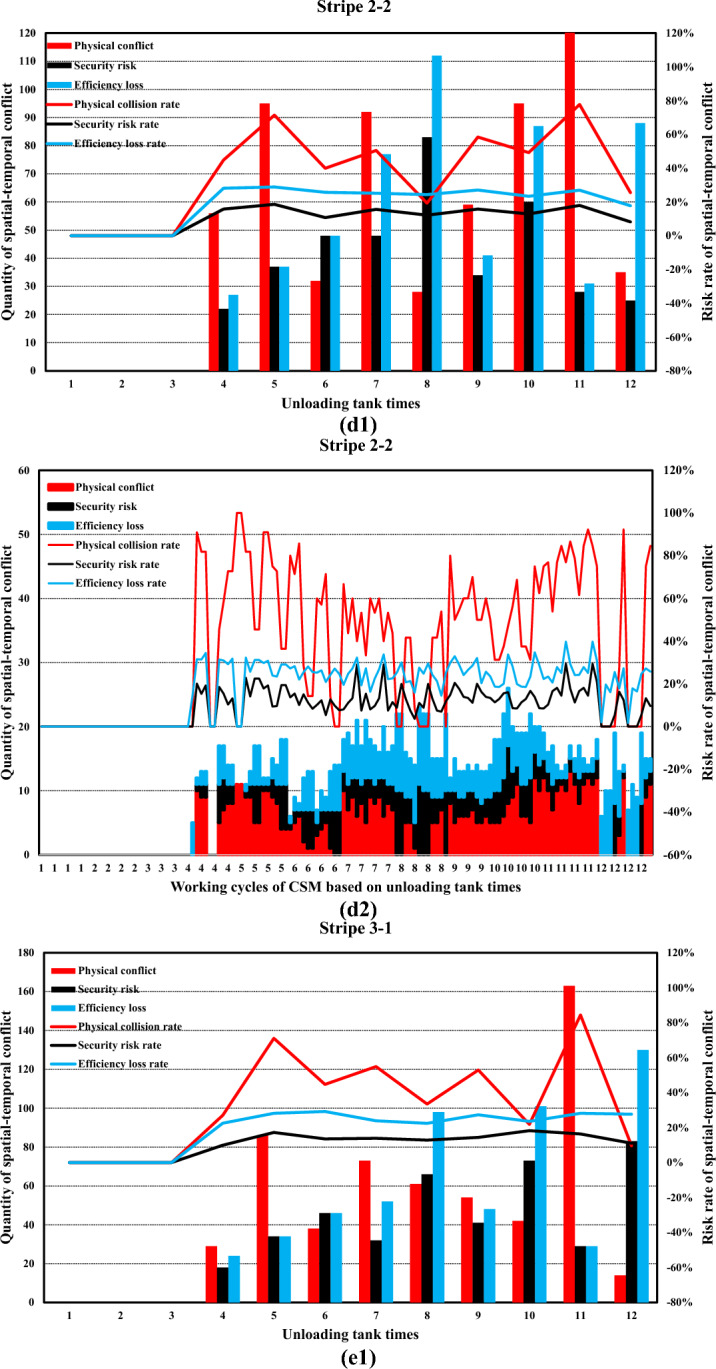

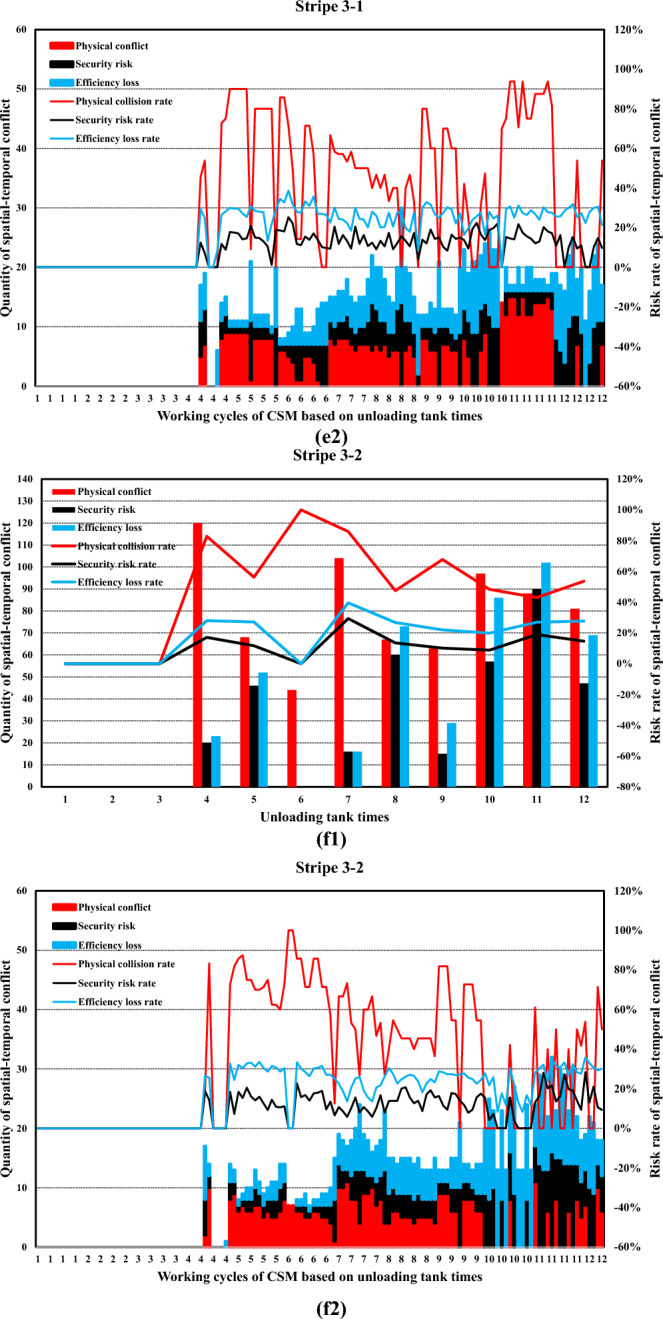
Statistical diagram of quantification results of the spatial–temporal conflict in the construction of each strip.

The average physical collision rate, security risk rate, and efficiency loss rate in the construction process of each zone and each tank, as well as the statistical results of each spatial–temporal conflict outbreak time, are obtained with simulation calculations. The average physical collision rate refers to the ratio between the number of physical collision accidents and the total number of micro steps. This paper mainly discusses the average physical collision rate of the construction process for the concrete material of a single tank and the single leveling process of a CSM. The average security risk rate and efficiency loss rate refer to the average value of the quantification results of spatial–temporal conflicts when all accidents of safety risk and efficiency loss occur. Figure 23 shows the statistical diagram of the quantification results of the spatial–temporal conflict in the construction of each strip, where a1–f1 show the spatial–temporal conflict results of the whole strip according to the decomposition of the strip, and a2–f2 show the spatial–temporal conflict results of the whole strip according to the amount of leveling work for the CSM, including forward or backward. The simulation results are as follows. In the simulation boundary conditions, the physical collision rate mainly occurs when two machines enter the field at the same time (the 4th and 5th tanks) in the initial period of construction. The physical collision incidents are up to 80 times, and the average physical collision rate reaches approximately 70%. In the middle of strip construction, the construction machinery starts to adjust the orientation (the 7th, 8th, and 9th tanks), the physical collision incidents can reach more than 140 times, and the average physical collision rate reaches about 90%. In the end period of construction, the construction area is further compressed (the 10th and 11th tanks), the physical collision incidents can reach up to 90 times, and the average physical collision rate reaches approximately 80%. Additionally, the condition of security risk and efficiency loss always exists, the security risk incidents reach more than 80 times, and the efficiency loss incidents reach more than 130 times.

#### Visualization of spatial–temporal conflict between construction machineries in the pouring block

The simulation and visualization system of the pouring construction considering the spatial–temporal conflict of construction machinery has the function of simulating the construction process and displaying the dynamic information of construction, and it can display the spatial–temporal conflict information obtained from the simulation calculation of the system in real time.

**Fig. 24:**
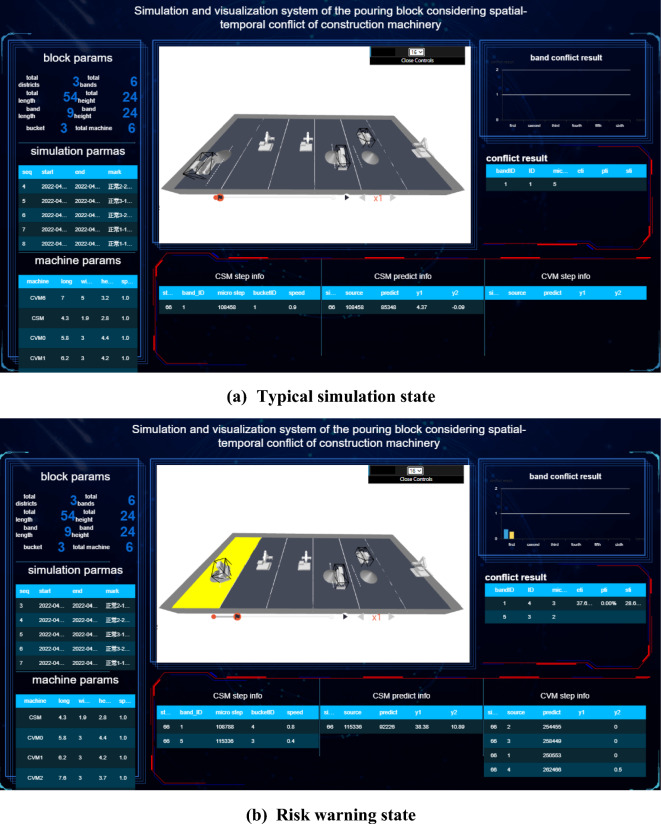

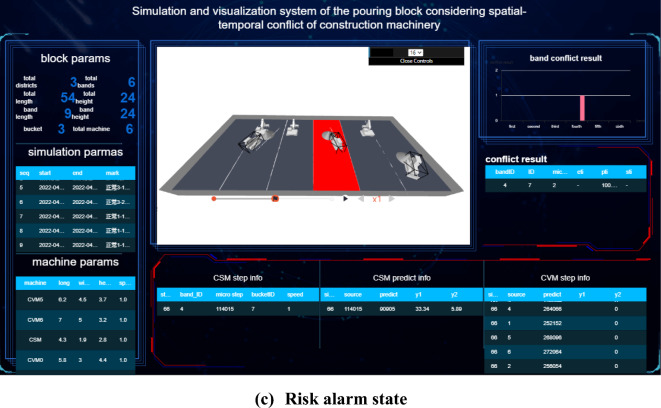
Simulation and visualization system of the pouring block considering the spatial–temporal conflict of construction machinery.

With the continuous progress of the simulation process, the dynamic simulation of the construction process evolves in the form of animation, and the construction information and the spatial–temporal conflict information of the construction process are displayed in real time. Figure 24a) shows the construction state, construction process, mechanical running track and micro-step track information, Fig. 24(b) shows the spatial–temporal conflict quantification information and conflict warning information, and Fig. 24(c) shows the conflict alarm information. The scheme rehearsal before pouring construction, the comprehensive analysis of the spatial–temporal conflict in the construction process, the warning and alarm of spatial–temporal conflict, and the construction scheme adjustment after the pouring construction process simulation can be realized based on the system simulation. Moreover, this can provide guidance for the actual pouring construction process, achieve the reasonable organization and scientific decision-making of the pouring construction process.

## Summary of results

The simulation results show that the physical collision accident mainly occurs in the leveling and vibrating process when concrete is unloaded 4–11 times for the typical zone, the security risk and efficiency loss caused by spatial–temporal conflict run through the whole strip construction process.

Meanwhile, the simulation results show that the physical collision rate mainly occurs when two machines enter the field at the same time (the 4th and 5th tanks) in the initial period of construction. The physical collision incidents are up to 80 times, and the average physical collision rate reaches approximately 70%. In the middle of strip construction, the construction machinery starts to adjust the orientation (the 7th, 8th, and 9th tanks), the physical collision incidents can reach more than 140 times, and the average physical collision rate reaches about 90%. In the end period of construction, the construction area is further compressed (the 10th and 11th tanks), the physical collision incidents can reach up to 90 times, and the average physical collision rate reaches approximately 80%. Additionally, the condition of security risk and efficiency loss always exists, the security risk incidents reach more than 80 times, and the efficiency loss incidents reach more than 130 times.

In view of the physical collision accidents caused by the spatial–temporal conflict in the strip construction process, the running track and state of the CSM and CVM entering the strip at the same time should be considered, and the approach time and running route should be reasonably adjusted. In addition, it is necessary to consider the movement trajectory of the construction machinery and the order of movement to reasonably avoid a conflict risk.

### The impact on practitioners

In the complex construction process of pouring blocks, the spatial–temporal conflict is one of the key factors restricting the acceleration of construction period and quality improvement. At present, the means of strengthening the construction site management by practitioners is to avoid the occurrence of safety accidents on the premise of reducing the construction efficiency. Meanwhile, in the high-intensity construction process, practitioners cannot effectively determine which stage has a greater risk of spatial–temporal conflict, which is not conducive to refined management. Thus, on the basis of the quantitative algorithm of spatial–temporal conflict of construction machinery, the simulation model of pouring construction system considering spatial–temporal conflict is constructed based on the discrete system theory. And the simulation and visual analysis software of the pouring construction system is developed, which can achieve the comprehensive analysis for the quantification of spatial–temporal conflict risks, conflict effects, and conflict information visualization. The aforementioned research has established a robust theoretical framework for advancing studies on intelligent planning and real-time control of collaborative trajectories in construction machinery. This foundation is crucial for achieving unmanned and intelligent pouring construction. Additionally, the research offers theoretical underpinnings for the quantitative assessment and analysis of spatial–temporal conflict risks during the pouring process. It serves as a valuable reference for the rational organization and scientific decision-making of pouring activities within construction blocks. Ultimately, this work introduces innovative ideas and methodologies aimed at enhancing the safety, efficiency, and precision of dam construction and management.

## Conclusion

In this research, the construction process of a pouring block in a concrete arch dam is taken as the research object. Based on the existing research on spatial–temporal conflicts in the construction process in engineering, research on the quantification method of the spatial–temporal conflict between the construction machinery in the pouring block and the simulation model of the conflict system is investigated. The quantification method and algorithm of the spatial–temporal conflict between construction machineries during the pouring process are proposed, which solves the problem of the current research on safety risks and efficiency losses in the field of engineering being biased toward empirical and index evaluation types. This provides theoretical support for intuitive and accurate evaluation and analysis of the risk of spatial–temporal conflict in the construction process. Furthermore, based on the model building and the quantification method of the spatial–temporal conflict between construction machineries, a simulation model of the pouring construction system considering the spatial–temporal conflict between construction machineries is finally established, which provides the model support and theoretical guidance for simulation analysis and research on self-adaptive adjustment of the conflict system in pouring block. Finally, taking the typical pouring block of the Baihetan arch dam as the research object, the system simulation boundary conditions and related construction parameters are determined. A simulation and visual analysis software of the pouring construction system considering the spatial–temporal conflict between construction machineries is developed, which can achieve the comprehensive analysis for the quantification of spatial–temporal conflict risks, conflict effects, and conflict information visualization.

The limitations and future research directions: (1) The interference from construction machinery systems (e.g., cable and tower cranes) during pouring operations has not been fully considered, and the simulation process remains oversimplified. Future work should establish a quantitative theoretical system encompassing spatial–temporal conflicts of such machinery. (2) While arch dams typically contain hundreds of concrete pouring blocks, the current simulation model has not comprehensively covered characteristic and construction information for all blocks. Future research should integrate this information to build a virtual analysis system for spatial–temporal conflicts during arch dam pouring construction. (3) Model validation tests for the construction system should be conducted through trajectory tracking and image analysis of construction machinery. Developing a multi-source construction data framework for collection, management, and analysis, an intelligent collaborative trajectory planning and real-time control system for construction machinery in the pouring blocks should be established based on the spatial–temporal conflict quantification method proposed in this study. Subsequent research should further explore unmanned and intelligent construction technologies for pouring blocks.

## Data Availability

The datasets used and/or analysed during the current study available from the corresponding author on reasonable request.
